# VHL loss enhances antitumor immunity by activating the anti-viral DNA-sensing pathway

**DOI:** 10.1016/j.isci.2024.110285

**Published:** 2024-06-15

**Authors:** Meng Jiao, Mengjie Hu, Dong Pan, Xinjian Liu, Xuhui Bao, Jonathan Kim, Fang Li, Chuan-Yuan Li

**Affiliations:** 1 Department of Dermatology, Duke University Medical Center, Durham, NC 27710, USA; 2 Department of Biochemistry, Molecular Cancer Research Center, School of Medicine, Sun Yat-sen University, Shenzhen, Guangdong, China; 3 School of Medicine, Duke University, Durham, NC 27710, USA; 4 Department of Pharmacology and Cancer Biology, Duke University Medical Center, Durham, NC 27710, USA

**Keywords:** Molecular biology, Immunity, Cell biology, Cancer

## Abstract

von Hippel-Lindau (*VHL*), known as a tumor suppressor gene, is frequently mutated in clear cell renal cell carcinoma (ccRCC). However, *VHL* mutation is not sufficient to promote tumor formation. In most cases other than ccRCC, *VHL* loss alters cellular homeostasis and causes cell stress and metabolic changes by stabilizing hypoxia-inducible factor (HIF) levels, resulting in a fitness disadvantage. In addition, the function of VHL in regulating immune response is still not well established. In this study, we demonstrate that *VHL* loss enhances the efficacy of anti-programmed death 1 (PD1) treatment in multiple murine tumor models in a T cell-dependent manner. Mechanistically, we discovered that upregulation of HIF1α/2α induced by *VHL* loss decreased mitochondrial outer membrane potential and caused the cytoplasmic leakage of mitochondrial DNA, which triggered cyclic GMP-AMP synthase-stimulator of interferon genes (cGAS-STING) activation and induced type I interferons. Our study thus provided mechanistic insights into the role of *VHL* gene loss in boosting antitumor immunity.

## Highlights

• VHL loss induced the cytoplasmic mitochondrial DNA leakage via HIF1/2-BNIP3 axis
• VHL loss caused cGAS-STING activation and type I interferon production
• cGAS-STING activation in VHL-deficient tumors facilitated ICB therapy

## Graphical abstract

**Figure:**
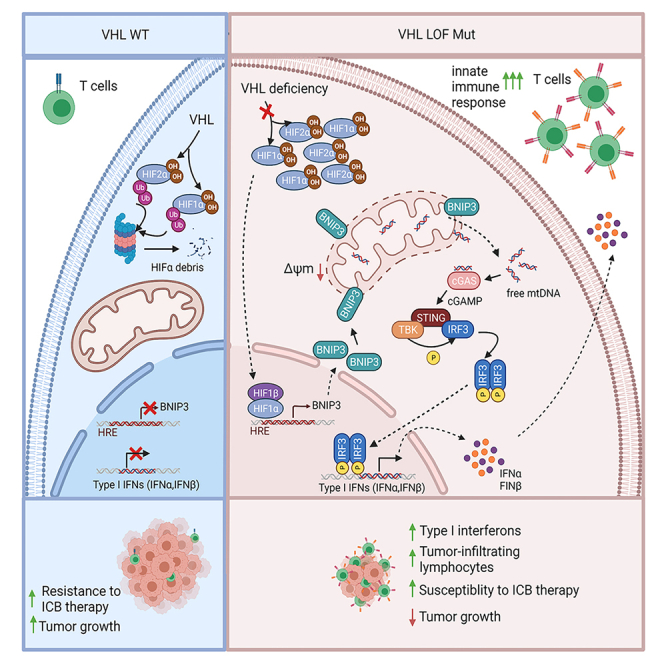

## Introduction

VHL (von Hippel-Lindau) is an E3 ligase responsible for regulating the cellular levels of hypoxia-inducible factors (HIFs),^1^^,^^2^^,^^3^ which are critical transcriptional factors regulating the cellular response to low oxygen tension.^4^^,^^5^ VHL is a recognized tumor suppressor, particularly in sporadic clear cell renal cell carcinomas (ccRCCs), where ∼91% of VHL mutations are found. Loss-of-function mutations can cause high HIF activities and increased vascular endothelial growth factor (VEGF) levels, which can promote renal cell carcinoma (RCC) tumor formation.^6^^,^^7^^,^^8^^,^^9^ However, VHL inactivation alone is insufficient for RCC development.^10^^,^^11^^,^^12^^,^^13^ In fact, VHL loss can lead to metabolic changes and create a fitness disadvantage in many cells.^14^^,^^15^^,^^16^ For example, HIF1α impairs cellular respiration and mitochondrial biogenesis in VHL-deficient cells.^14^ Loss of VHL also increases the expression of cyclin kinase inhibitors p21 and p27 that cause cell arrest and/or senescence.^17^^,^^18^

VHL plays a crucial role in regulating the immune system through its regulation of the stable-state level and activities of HIFs.^19^^,^^20^^,^^21^ For instance, loss of VHL stabilizes and increases HIF activity, resulting in enhanced CD8^+^ T lymphocyte cytotoxicity.^20^ In addition, VHL loss-mediated HIF activation leads to changes in gene expression that can significantly impact the tumor immune microenvironment (TIME) with regard to both the composition and the function of the immune cells.^22^ HIF activation can be a double-edged sword that may reduce T cell differentiation while also enhancing T cell proliferation and activation.^20^^,^^23^ Moreover, the activity of HIFs affects cytokine production, which can reprogram immune responses. For example, HIF activation in T cells is associated with the upregulation of certain pro-inflammatory cytokines and cytolytic molecules, such as interferon-gamma (IFNγ), granzymes, and tumor necrosis factors, thereby stimulating cytotoxic T cell responses and impacting the effectiveness of immune checkpoint blockade (ICB) therapy.^20^^,^^24^^,^^25^

ICB therapy has emerged as a potent tool in the fight against various types of cancer in recent years, including melanoma and ccRCC.^26^ The existing paradigm for determining the efficacy of cancer immunotherapy in a given malignancy centers on multiple intra- and intercellular factors, such as gene mutations, antigen presentation, and the diversity of major histocompatibility complex (MHC) as well as T cell receptor (TCR) repertoires.^27^ Tumor mutation burden (TMB) is a crucial parameter for predicting tumor responses to immunotherapy.^28^^,^^29^^,^^30^ The main reasoning is that a higher TMB leads to higher numbers of neoantigens that are likely to be recognized by the host’s T cells, which is critical for the success of ICB therapy.^31^ However, it cannot explain the responsiveness of ccRCC to ICB therapy, where the TMB of ccRCC is only very moderate.^28^ A previous study has implicated that endogenous retrovirus activation may be involved in ccRCC response to ICB.^32^ In view of the prevalence of VHL mutations in ccRCC, we focus on *VHL* gene to examine in detail its potential roles in modulating tumor responses to ICB therapy.

## Results

### *Vhl* deficiency increased susceptibility to ICB therapy in murine tumor models

**Figure 1:**
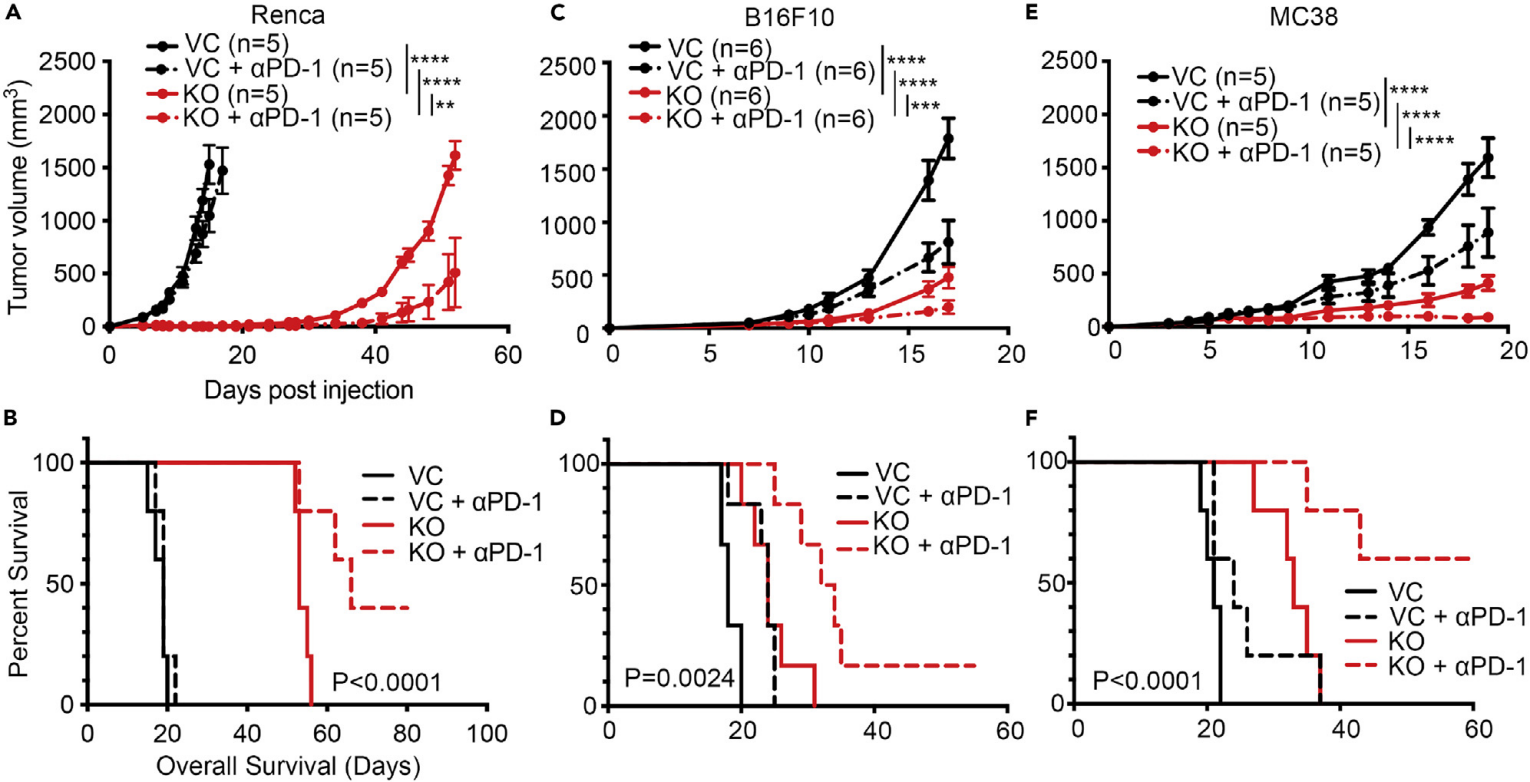
*VHL* loss enhances murine tumor responses to ICB therapy (A and B) Tumor growth (A) and Kaplan-Meier analysis (B) of BALB/c mice subcutaneously inoculated with 1 × 10^6^ VC or *Vhl*-KO Renca cells (*n* = 5) and treated with isotype control or an αPD-1 antibody. About 100 μg isotype control or αPD-1 antibodies were injected intraperitoneally (i.p.) on days 5, 8, and 11 post tumor cell inoculation. (C and D) Tumor growth (C) and Kaplan-Meier survival analysis (D) of C57BL/6J mice subcutaneously inoculated with 1 × 10^5^ VC or *Vhl*-KO B16F10 cells (*n* = 5) and treated with isotype control or an αPD-1 antibody. About 100 μg isotype control or αPD-1 antibodies were injected i.p. on days 7, 10, 13, and 16 post tumor cell inoculation. (E and F) Tumor growth (E) and Kaplan-Meier analysis (F) of C57BL/6J mice subcutaneously inoculated with 5 × 10^5^ VC or *Vhl*-KO MC38 cells (*n* = 5) and treated with isotype control or αPD-1 antibodies. About 100 μg isotype control or αPD-1 antibodies were injected i.p. on days 6, 9, and 12 post tumor cell inoculation. Error bars represents mean ± SEM. ∗*p* < 0.05; ∗∗*p* < 0.01; ∗∗∗*p* < 0.001; ∗∗∗∗*p* < 0.0001; n.s. not significant.

To evaluate the relevance of *Vhl* gene loss in ICB therapy, we conducted anti-programmed death 1 (αPD1) therapy in several *Vhl*-deficient tumor lines following the schedule depicted in Figure S1D. In the murine RCC Renca tumor model, *VHL* loss alone suppressed tumor growth (Figures S1A and 1A). This result is consistent with a published study demonstrating that *VHL* deletion in Renca cells restrains tumor growth *in vivo* using mouse models.^33^ Moreover, αPD1 treatment had minimal effects in control tumors but synergized with *Vhl* gene knockout (KO), significantly suppressing tumor growth (Figure 1A) and extending the survival of tumor-bearing mice (Figure 1B). Indeed, 2/5 mice in the αPD1-treated *Vhl*-KO group remained long-term survivors, indicating significant synergy between *Vhl* gene loss and ICB therapy. In the B16F10 melanoma line, *Vhl* gene loss (Figure S1B) also significantly attenuated tumor growth in immunocompetent C56BL/6J mice (Figure 1C). Moreover, αPD1 treatment significantly suppressed the growth of *Vhl-KO* tumors (Figures 1C and 1D), with 1/6 mice remaining tumor-free in the entire period of observation (Figure 1D). We further examined the efficacy of ICB therapy in *Vhl-KO* MC38 tumors (Figure S1C). Our data indicated that αPD1 treatment caused additional growth delay in *Vhl-KO* MC38 tumors, which were already substantially slower than control tumors (Figures 1E and 1F). Notably, 3/5 mice in the αPD1-treated *Vhl-KO* group were long-term survivors (Figure 1F). To rule out the possibility that the off-target effects of CRISPR-Cas9 were responsible for the behavior of the *Vhl-KO* tumors, we restored *Vhl* expression by ectopic gene transduction (Figure S1E). Our data showed that ectopic expression of wild-type (WT) mouse *Vhl* (*mVhl*) in *Vhl-KO* MC38 cells abrogated the delay in tumor formation from the latter (Figures S1F and S1G), thereby supporting an essential role for VHL in this process. Our results, therefore, suggest that *VHL* loss significantly attenuated tumor growth and made them more susceptible to ICB therapy in multiple murine tumor models.

### *VHL* deficiency stimulates intratumoral lymphocyte infiltration

**Figure 2:**
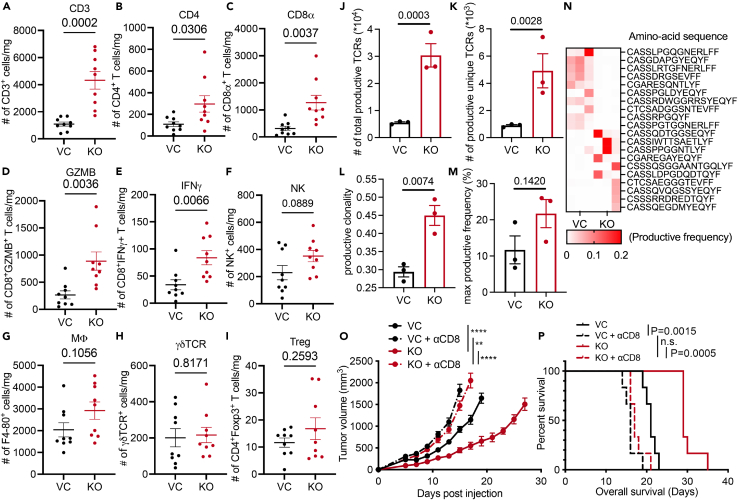
*VHL* loss increases intratumoral lymphocyte infiltration (A–I) Flow cytometry profiling of intratumoral lymphocytes in control and *VHL*-KO MC38 tumors grown in syngeneic C57BL/6 mice. Mice bearing VC or *Vhl*-KO MC38 tumors (*n* = 9) were euthanized on day 15 post tumor cell inoculation. The numbers of immune effector cells per mg of tumor were then obtained using flow cytometry. Error bars represents mean ± SEM. (J–N) ImmunoSEQ analysis of TCRβ repertoire. Total productive TCRs (J), productive unique TCRs (K), productive clonality (L), and maximum productive frequency (M) were shown for control and *Vhl*-KO tumors. Heatmap (N) showing the top 10 (5%) most frequent productive TCR sequences in VC and *Vhl*-KO tumors. (O and P) *In vivo* tumor growth and Kaplan-Meier survival analysis of C57BL/6J mice bearing VC and *Vhl*-KO MC38 tumors (*n* = 6) following depletion of CD8^+^ T cells using anti-CD8 antibodies. Each mouse received i.p. injection of 100 μg isotype or αCD8b antibodies on days 1, 4, and 7 post tumor cell inoculation. Error bars represents mean ± SEM. ∗*p* < 0.05; ∗∗*p* < 0.01; ∗∗∗*p* < 0.001; ∗∗∗∗*p* < 0.0001; n.s. not significant.

The substantial tumor growth delay caused by *VHL* gene loss and its synergy with αPD1 treatment led us to posit that *VHL* played a critical role in regulating the TIME. We used flow cytometry to analyze intratumoral infiltration of lymphocytes in control and *Vhl-KO* MC38 tumors to examine our hypothesis (Figure S2A). Our results show significantly more CD3^+^ T cells in *VHL-KO* than in control MC38 tumors (Figure 2A). The increase was observed in both CD4^+^ T and CD8^+^ T subsets (Figures 2B and 2C). Notably, both the numbers of GZMB^+^ CD8^+^ T cells and IFNγ^+^ CD8^+^ T cells were significantly increased (Figures 2D and 2E), indicating heightened activation of cytotoxic T lymphocytes in *Vhl-KO* tumors. Our analysis also showed high natural killer (NK^+^) cells in Vhl-KO tumors that did not reach statistical significance (Figure 2F). In contrast, the numbers of F4-80^+^ macrophages (Mφ), γσTCR^+^ T cells, and Foxp3^+^ Tregs were comparable between control and *Vhl-KO* tumors (Figures 2G–2I).

To further characterize the effect of *Vhl-KO* on intratumoral T cells, we analyzed the TCR repertoire of control and *Vhl*-KO MC38 tumors using the ImmunoSEQ approach.^34^ Our analysis indicated that the numbers of both total productive TCRs (Figure 2J) and productive unique TCRs (Figure 2K) were significantly increased in *Vhl-KO* tumors, suggesting substantially more T cells with increased TCR diversity. Furthermore, productive clonality, an index representing the diversity of rearranged T cell clones, increased substantially in *Vhl-KO* MC38 tumors (Figure 2L). Since each distinct TCR clonal expansion can be regarded as a result of T cell proliferation and activation in response to the specific recognition of tumor antigen fragments, higher productive clonality in *Vhl-KO* MC38 tumors suggests the activation of different T cell clonotypes that are likely to target a higher number of different tumor-specific antigens. Moreover, the maximum productive frequency measuring the frequency of dominant TCR clones also trended higher in *Vhl-KO* MC38 clones, despite not reaching statistical significance (Figure 2M). Finally, the predominant T cell clones in *Vhl-KO* tumors had different TCR sequences than those in the control tumors (Figure 2N), suggesting activation of different T cell subsets targeting different tumor antigens between *VHL*-WT and *Vhl*-KO tumors.

To evaluate the relative contribution of different immune effector subsets involved in antitumor immunity, we used antibodies to deplete CD4^+^ T cells, CD8^+^ T cells, and NK cells in mice bearing control and *Vhl-KO* MC38 tumors and observed their growth kinetics. Administration of an αCD8 antibody significantly accelerated the growth of vector control (VC) and *Vhl-KO* tumors and completely abrogated the substantial growth delay of *Vhl*-KO tumors (Figures 2O and 2P). On the other hand, administration of αCD4 and αNK1.1 antibodies did not affect tumor growth compared to those receiving isotype controls (Figures S2B–S2E). Therefore, our data suggest a pivotal role for CD8^+^ T cells in mediating the growth delay in tumors with *Vhl* gene loss.

### *VHL* deficiency causes constitutive activation of type I interferons

**Figure 3:**
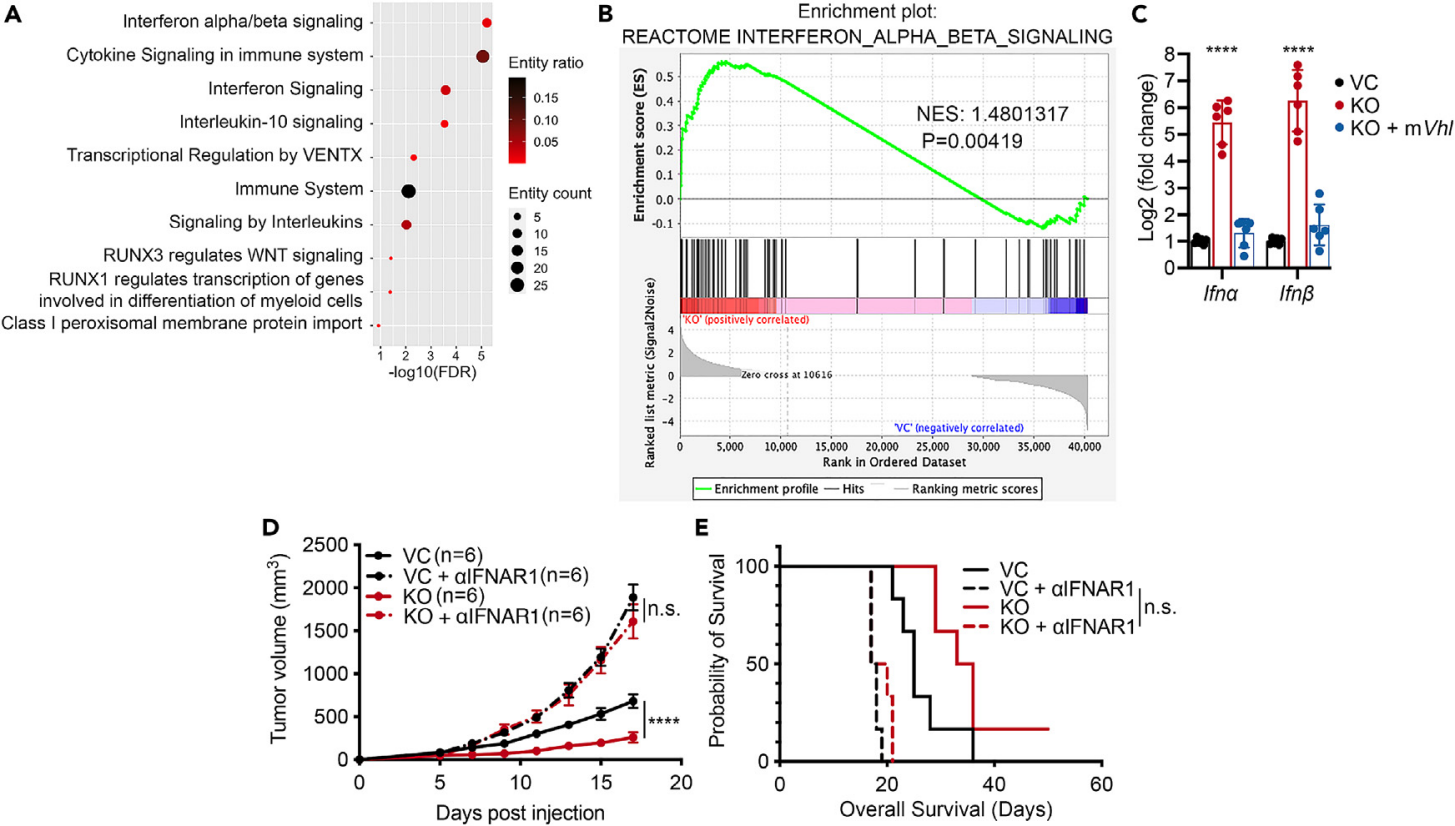
Type I interferon production is responsible for *VHL*-KO-induced tumor growth suppression (A) Gene Ontology (GO) analysis shows top-10 pathways enriched in 786-O cells compared to 786-O cells with restored wild-type human *VHL* (*hVHL*) expression using publicly available data (accession number: GSE108229). (B) GSEA score curve plot showing the IFNα/β signaling pathway enriched in *VHL*-KO Caki-1 cells. NES, normalized enrichment score. (C) Quantitative reverse-transcription PCR (RT-qPCR) analysis of mRNA levels of *Ifnα* and *Ifnβ* in VC, *Vhl*-KO MC38 cells, and *Vhl*-KO MC38 cells with an exogenously expressed wild-type mouse *VHL* (*mVhl*) gene. We performed three independent experiments. Error bars represent mean ± SD. (D and E) Tumor growth and Kaplan-Meier survival analysis of C57BL/6J mice bearing VC and *Vhl*-KO MC38 tumors (*n* = 6) following αIFNAR1 antibody treatments to suppress type I interferon signaling. About 100 μg isotype or αIFNAR1 antibodies per mouse were administered i.p. on days 5, 8, and 11 post tumor cell inoculation. Error bars represent mean ± SEM. *p* < 0.05 is considered statistically significant. ∗*p* < 0.05; ∗∗*p* < 0.01; ∗∗∗*p* < 0.001; ∗∗∗∗*p* < 0.0001; n.s. not significant.

To unravel the molecular mechanism of how *VHL* loss stimulates the antitumor immune response, we analyzed a publicly available dataset (accession number: GSE108229) comparing the transcriptional differences between parental 786-O RCC cells that carry a homozygous nonsense mutation in the *VHL* gene and 786-O cells with ectopic human *VHL* (*hVHL*) expression.^35^ Consistent with our findings from *in vivo* tumor growth delay studies, Gene Ontology analysis showed that *VHL* deficiency led to the activation of multiple immune-stimulating pathways (Figure 3A). Notably, we found that IFNα/β signaling is highly upregulated in *VHL*-deficient 786-O cells (Figure 3A). To further validate this finding, we generated *VHL-KO* Caki-1 cells, a human clear cell carcinoma cell line with WT *VHL* expression (Figures S3A and S3B). We then carried out RNA sequencing (RNA-seq) to identify transcriptomic changes in *VHL-KO* Caki-1 cells. Principal component analysis of the RNA-seq data demonstrated significant changes between VC and *VHL-KO* Caki-1 cells (Figure S3C). Furthermore, gene set enrichment analysis (GSEA) identified significant enrichment of gene signatures involving IFNα/β signaling and positive regulation of T cell-mediated cytotoxicity in *VHL-KO* Caki-1 cells (Figures 3B and S3D), consistent with our findings in 786-O cells and intratumoral lymphocytes infiltration in MC38 tumors. IFNα/β are type I interferons (IFNs) that can upregulate antigen presentation on the surface of tumor cells, which in turn stimulates the cross-presentation of tumor-specific antigens by professional antigen presentation cells such as macrophages and dendritic cells, which are essential for antitumor immunity.^36^ Indeed, flow cytometry analysis showed a higher level of mouse H2K^b^/H2D^b^ expression on the surface of *Vhl-KO* MC38 cells compared to control MC38 cells (Figures S3D and S3E). Furthermore, the mRNA levels of multiple human MHC class I (human leukocyte antigen) proteins were also substantially higher in *VHL-KO* Caki-1 cells (Figure S3F). These data thus provide strong evidence indicating a vital role for *VHL* deficiency in stimulating the type I IFNs.

To further characterize the involvement of IFNα/β signaling in suppressing the growth of *VHL-KO* murine tumors, we examined the expression of *Ifnα* and *Ifnβ* in VC and *Vhl-KO* MC38 cells, and *Vhl-KO* MC38 cells with restored *mVhl* gene expression. *Ifnα/β* mRNA levels increased by approximately 32-fold and 64-fold in *Vhl-KO* MC38 cells, respectively. Notably, expression levels of both *Ifnα/β* reverted to control levels with the restoration of *mVhl* expression in *Vhl-KO* MC38 cells (Figure 3C). To determine the functional importance of IFNα/β signaling in regulating antitumor immunity *in vivo*, we conducted a tumor growth delay experiment where we blocked the type I IFN signaling *in vivo* by injecting an αIFNAR1 antibody, which blocks the receptor for both IFNα/β. Strikingly, blocking IFNAR signaling abrogated the tumor growth delay in *Vhl-KO* MC38 cells. Moreover, tumor growth from *Vhl-KO* MC38 tumors in mice receiving the αIFNAR1 antibody was even faster than that of control tumors, similar to VC MC38 tumors treated with the αIFNAR1 antibody (Figures 3D and 3E). These results suggest that type I IFN signaling played an essential role in *Vhl-KO*-mediated antitumor immunity.

### Constitutive activation of the cGAS-STING pathway responsible for *VHL* loss-induced type I IFN activation

**Figure 4:**
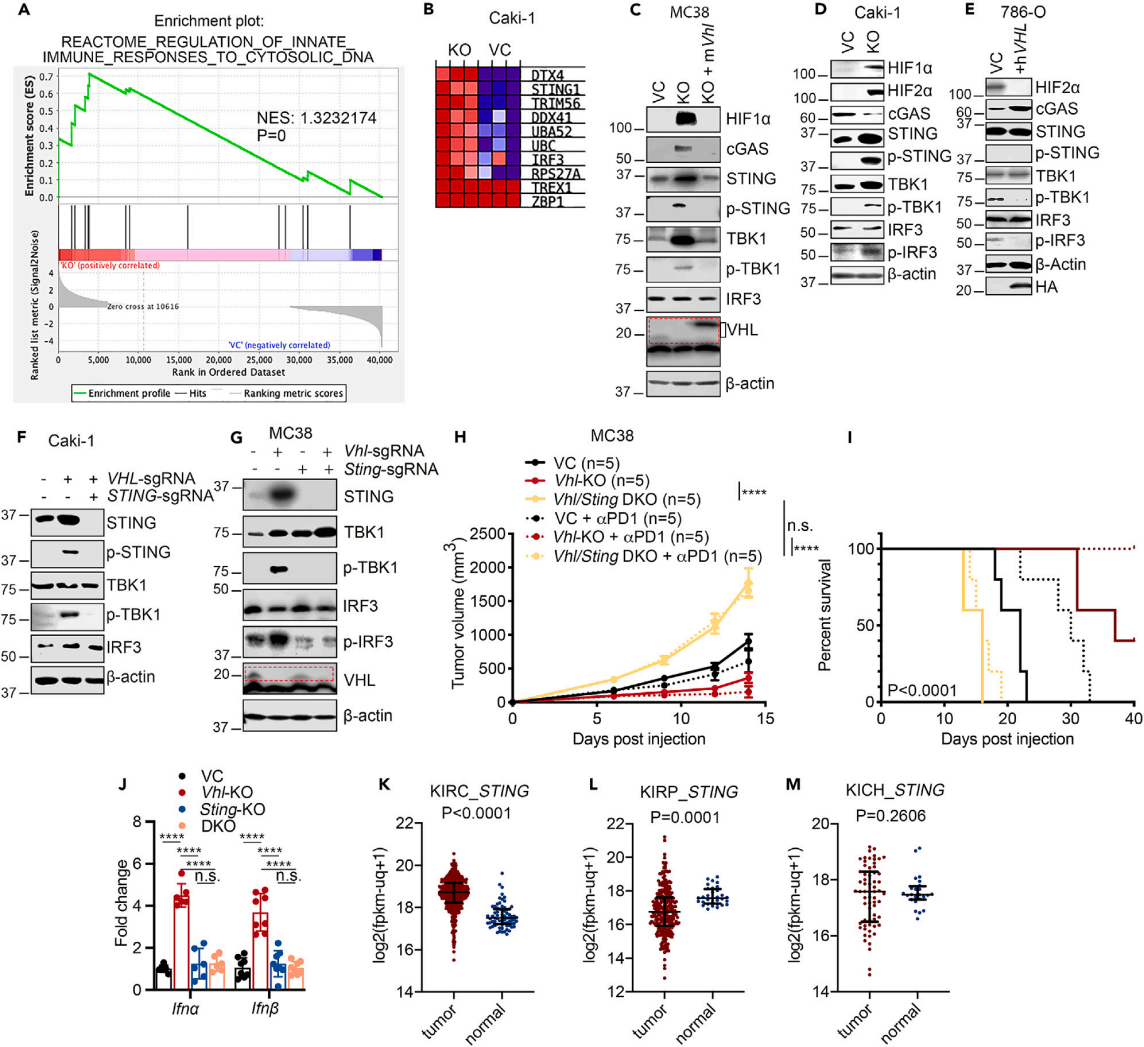
Activation of cGAS-STING signaling in *VHL*-KO cells and ccRCC human tumor tissues (A) GSEA analysis of control and *VHL*-KO Caki-1 cells indicating elevated expression of genes involved in sensing cytosolic DNA. (B) Heatmap of top-10 differently expressed genes involved in regulating innate immune response to cytosolic DNA in control and *VHL*-KO Caki-1 cells. (C and D) Western blot analysis examining the effect of *Vhl/VHL* gene loss on cGAS-STING signaling and its downstream effectors in MC38 cells (C) and Caki-1 cells (D). In (C), ectopic m*Vhl* was re-expressed in *Vhl*-KO MC38 cells. (E) Western blot analysis evaluating the effect of the ectopic expression of h*VHL* on cGAS-STING signaling in *VHL*-deficient 786-O cells. (F and G) Immunoblot analysis of that STING and TBK1 activation in *VHL*/*STING* DKO Caki-1 (F) and MC38 (G) cells. (H) Quantitative PCR determination of *Ifnα/β* levels in *Vhl*-KO, *Sting*-KO, and *Vhl*/*Sting* DKO MC38 cells. (I and J) Tumor growth (I) and Kaplan-Meier analysis (J) of C57BL/6 mice subcutaneously inoculated with VC, *Vhl*-KO, or *Vhl/Sting* DKO MC38 cells and treated with isotype control or αPD-1 antibody (100 μg per mouse) on days 6, 9, and 12 post tumor cell inoculation. *n* = 5 per group. (K–M) Comparisons of *STING* RNA expression levels between tumor tissues and adjacent normal tissues from TCGA_KIRC (clear cell renal cell carcinoma) (K), TCGA_KIRP (kidney renal papillary cell carcinoma) (L), and TCGA_KICH (kidney chromophobe renal cell carcinoma) (M) cohorts. *STING* expression is only significantly higher in ccRCC (KIRC) vs. adjacent tissues where VHL is mutated in most tumors. Error bars represent mean ± SD. *p* < 0.05 is considered statistically significant. ∗*p* < 0.05; ∗∗*p* < 0.01; ∗∗∗*p* < 0.001; ∗∗∗∗*p* < 0.0001; n.s. not significant.

To determine the mechanism of type I IFN induction in *VHL-KO* cells, we further examined RNA-seq data of *VHL-KO* Caki-1 cells using GSEA analysis. Our analysis demonstrated that *VHL* deficiency induced the expression of genes associated with the cyclic GMP-AMP synthase (cGAS)-stimulator of interferon genes (STING) pathway involved in sensing the cytoplasmic presence of double-stranded DNA (dsDNA) (Figures 4A and 4B). The cGAS-STING pathway activation can induce interferon-stimulated genes and type I IFN production.^37^^,^^38^^,^^39^ Based on this analysis, we conducted immunoblot analysis of proteins associated with the VHL protein and the cGAS-STING pathway. As expected, we found that *VHL* loss in MC38 and Caki-1 cells caused the accumulation of HIF1 and 2α (Figures 4C and 4D). In addition, it upregulated the phosphorylated STING in both cell lines (Figures 4C and 4D), consistent with our RNA-seq analysis. Interestingly, we also found an elevation in the total protein level of STING in all *VHL*-deficient cells. The cGAS expression was increased in *Vhl*-KO MC38 cells but reduced in *VHL*-KO Caki-1 cells. This is likely due to the cell variation between mouse and human lines (Figures 4C and 4D). Therefore, VHL may also regulate cGAS/STING protein levels through a mechanism that is not fully understood at present. Furthermore, *VHL* loss activated several downstream effectors of the cGAS-STING axis, including the TANK-binding kinase 1 (TBK1), interferon regulatory factor 3 (IRF3), and their phosphorylated forms, consistent with the activation of the cGAS-STING pathway (Figures 4C and 4D). Furthermore, the observation of *VHL* gene loss-induced TBK1 activation was consistent with a recently published study.^40^ In addition, we also observed a similar relationship between *Vhl*-KO and activation of the cGAS-STING pathway in B16F10 melanoma cells (Figure S4A). Moreover, to further establish the relationship between *VHL* loss and activation of the cGAS-STING axis, we re-expressed *mVhl* and *hVHL* in *Vhl-KO* mouse MC38 cells and *VHL-*deficient human 786-O cells, respectively (Figures 4C and 4E). Immunoblot analysis showed that the restoration of *VHL* abrogated the activation of STING, TBK1, and IRF3 in *VHL*-deficient cells (Figures 4C and 4E).

To determine if the cGAS-STING pathway is essential for *VHL* gene loss-induced IFNα/β production, we generated *VHL/STING* double-KO (*DKO*) cells for Caki-1 and MC38 cells, respectively (Figures 4F and 4G). Immunoblot analysis showed *STING-KO* abrogated *VHL* loss-induced type I IFN response in Caki-1 (Figure 4F) and MC38 (Figure 4G) cells. In contrast, genetic KO of *Mda5*, a factor involved in sensing the cytoplasmic presence of double-stranded RNA, did not affect *Vhl-KO*-induced STING and TBK1 activation (Figure S4B). In addition, *Sting-KO* eliminated *Vhl-KO*-dependent *Ifnα* and *Ifnβ* mRNA upregulation (Figure 4H), suggesting the cGAS-STING-TBK1 axis is functionally responsible for *VHL* loss-induced type I IFN response. Notably, *Vhl/Sting* DKO completely reversed tumor growth delay and sensitization to anti-PD1 therapy caused by *Vhl* loss (Figures 4I and 4J). Furthermore, the increased intratumoral lymphocyte infiltration observed in *Vhl*-KO tumors, especially those of CD3^+^ T, CD8α^+^ T, and CD8^+^ GZMB^+^ T cells, was significantly reduced in *Vhl/Sting* DKO tumors (Figures S4C–S4H), further supporting the key role of cGAS-STING in *Vhl*-deficient tumor sensitivity to anti-PD1 blockades.

We also determined *STING* mRNA expression levels in three human The Cancer Genome Atlas (TCGA) RCC cohorts: kidney renal clear cell carcinoma (KIRC), kidney renal papillary cell carcinoma (KIRP), and kidney chromophobe renal cell carcinoma (KICH). Compared with *STING* expression levels in adjacent normal tissues, only KIRC, representing ccRCC, showed significantly higher expression levels (Figure 4K). In contrast, KIRP (Figure 4L) showed lower expression levels than normal tissues, while KICH showed no difference (Figure 4M). Because among three types of RCC, VHL function is lost in most KIRC but not in KICH or KIRP, the human RCC *STING* expression data were thus consistent with our preclinical observation.

### Mitochondrial dysfunction and cytoplasmic leakage of mtDNA responsible for *VHL* deficiency-induced cGAS-STING activation

**Figure 5:**
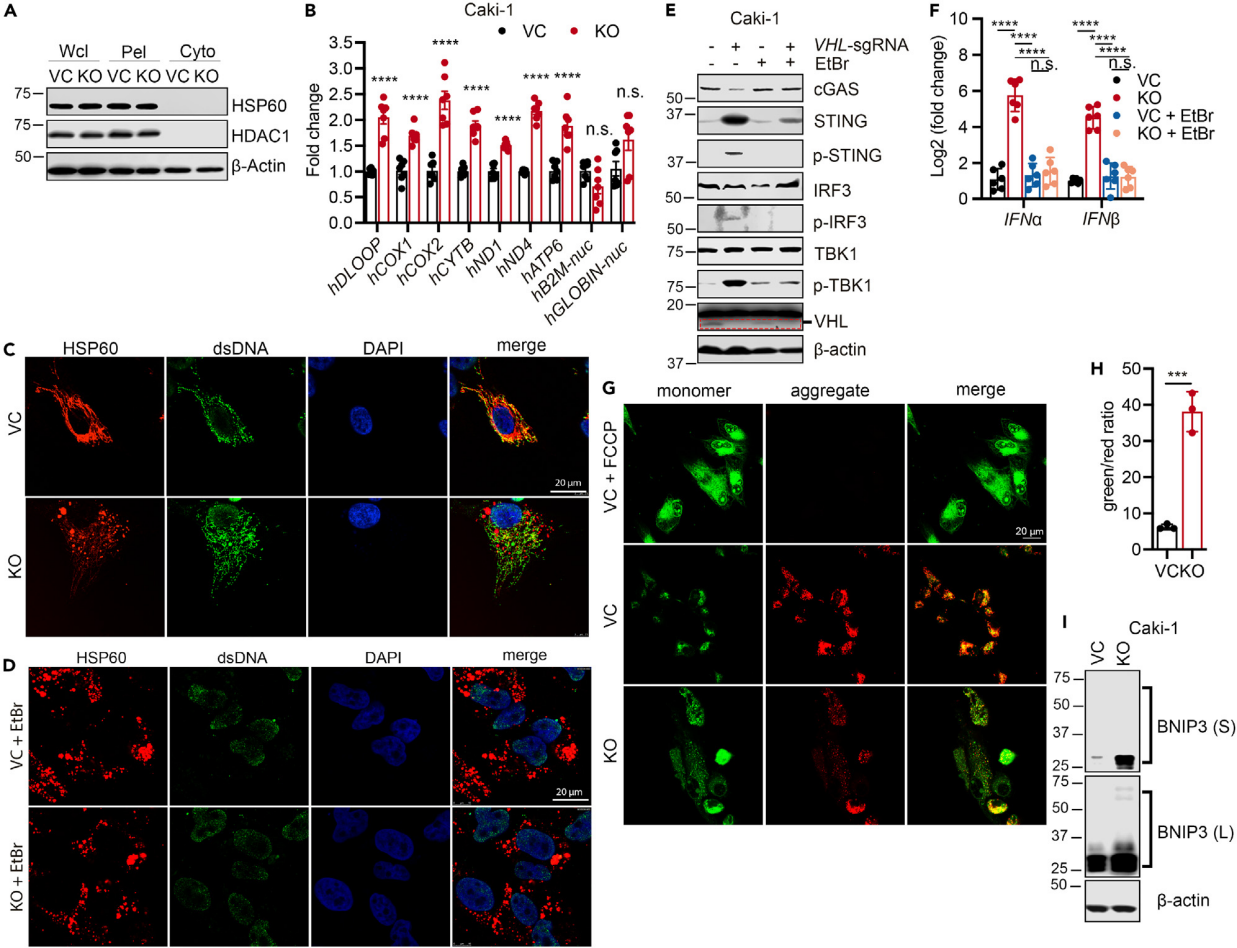
Cytoplasmic leakage of mtDNA as the key driver of cGAS-STING activation in *VHL*-deficient cancer cells (A) Immunoblot analysis validation of the absence of nuclear (*HDAC1* as the marker) and mitochondrial (*HSP60* as the marker) proteins. Wcl, whole cell lysate; Pel, pellet fraction; Cyto, cytosolic fraction. (B) RT-qPCR measurement of mtDNAs in the cytosols of VC and *VHL*-KO Caki-1 cells (*n* = 3). Error bars represent mean ± SEM. (C) Immunofluorescent localization of the mitochondria (as detected by HSP60) and dsDNA in the cytoplasm of VC and *VHL*-KO Caki-1 cells. Scale bar, 20 μm. (D) Immunofluorescence detection of cytoplasmic dsDNA in VC and *VHL*-KO Caki-1 cells after a 21-day treatment in 100 ng/mL EtBr to deplete mtDNA. Scale bar, 20 μm. (E) Immunoblot analysis of proteins involved in cGAS-SITNG signaling in VC and *VHL*-KO Caki-1 cells treated with vehicle or 100 ng/mL EtBr for 21 days. (F) RT-qPCR analysis of the mRNA levels of *IFNα* and *IFNβ* in VC and *VHL*-KO Caki-1 cells treated with vehicle or 100 ng/mL EtBr for 21 days (*n* = 6). Error bars represent mean ± SD. (G and H) Mitochondrial membrane potential (MtMP) characterization by JC-1 staining in VC and *VHL*-KO Caki-1 cells as detected by immunofluorescence microscopy (G) and flow cytometry (H). Scale bar, 20 μM. Error bars represent mean ± SD. ∗*p* < 0.05; ∗∗*p* < 0.01; ∗∗∗*p* < 0.001; ∗∗∗∗*p* < 0.0001; n.s. not significant. (I) Immunoblot analysis of BNIP3 expression in VC and *VHL*-KO Caki-1 cells.

We next attempted to determine the source of the cytosolic dsDNA that triggers cGAS-STING activation. We first fractionated VC and *VHL-KO* Caki-1 cellular lysates into pellet and cytosolic fractions and verified the success of the fractionation by using HSP60 and HDAC1 as markers for mitochondrial and nuclear fractions, respectively^41^^,^^42^^,^^43^ (Figure 5A). We then purified DNA from the cytosolic fractions and performed quantitative PCR analysis using primers that amplify nuclear (nucDNA) and mitochondrial (mtDNA)-encoded genes. Our results indicate no significant differences in the amount of cytosolic nucDNA between VC and *VHL-KO* Caki-1 cells (Figure 5B). In contrast, we consistently detected more mtDNA in the cytosolic fraction of *VHL-KO* than VC Caki-1 cells (Figure 5B), strongly suggesting mtDNA leakage into the cytoplasm as the trigger for cGAS-STING activation in *VHL-KO* cells. We also made similar observations in murine MC38 and B16F10 tumor cells (Figures S5A–S5D). We next attempted to detect the cytoplasmic presence of dsDNA by immunofluorescence staining in VC and *VHL-KO* Caki-1 cells. Using an antibody specific for dsDNA, we observed that most cytoplasmic DNA was co-localized with HSP60 in the mitochondria in control cells (Figure 5C). However, a substantial amount of dsDNA did not co-localize with HSP60 in *VHL-KO* cells (Figures 5C and S5E), suggesting extra-mitochondrial locations for the dsDNA. These data thus provide correlative evidence that mtDNA leaked into the cytoplasm in *VHL-KO* cells is responsible for the observed cGAS-STING activation. To determine if mtDNA is functionally required for cGAS-STING activation, we used an established protocol to deplete mtDNA by ethidium bromide (EtBr).^44^^,^^45^^,^^46^ After a 21-day EtBr treatment, we could deplete most cytosolic mtDNA as confirmed by immunostaining using an anti-dsDNA antibody in Caki-1(Figure 5D) and MC38 (Figure S5F) cells. Our data suggest that EtBr treatment substantially suppressed cGAS-STING and/or type I IFN response *VHL-KO* Caki-1 (Figure 5E) and MC38 (Figures 5F and S5G) cells. These results, therefore, support the notion that mtDNA leakage into the cytoplasm by *VHL-KO* is responsible for activating the cGAS-STING pathway and inducing type I IFN production.

Since mtDNA leakage into the cytoplasm is most likely due to mitochondria damage, we sought to understand how *VHL* deficiency may disrupt mitochondrial functions. mtDNA leakage is usually associated with lowered mitochondrial membrane potential (MtMP), closely associated with the permeability changes in mitochondrial membranes.^47^ To determine whether *VHL* deficiency influences mitochondrial membrane permeability, we assessed mitochondrial permeability by using the JC-1 fluorescent probe^48^^,^^49^ in VC and *VHL-KO* cells. Flow cytometry analysis found a significantly higher green(monomer)/red(aggregate) fluorescence ratio of JC-1 in *VHL-KO* cells, indicating significantly lowered MtMP in VHL-deficient Caki-1 cells (Figures 5G, 5H, and S6A) and MC38 cells (Figures S6B–S6D). These data suggest that *VHL* deficiency causes a significant reduction in MtMP. How does *VHL* deficiency cause a decrease in MtMP? Previous studies have shown that hypoxia can induce the expression of BCL2 interacting protein 3 (BNIP3),^50^^,^^51^ which can increase mitochondrial outer membrane permeability and decrease membrane potential.^52^^,^^53^ Therefore, we hypothesized that *VHL* deletion was akin to subjecting cells to a condition of perpetual hypoxia by permanently upregulating HIFα expression, which could upregulate BNIP3 expression. Indeed, our data indicate that *VHL* KO significantly increased the level of BNIP3 (Figure 5I), consistent with our hypothesis.

### Increased HIF1/2α and BNIP3 levels in *VHL*-deficient cells are responsible for mitochondrial dysfunction and mtDNA leakage

**Figure 6:**
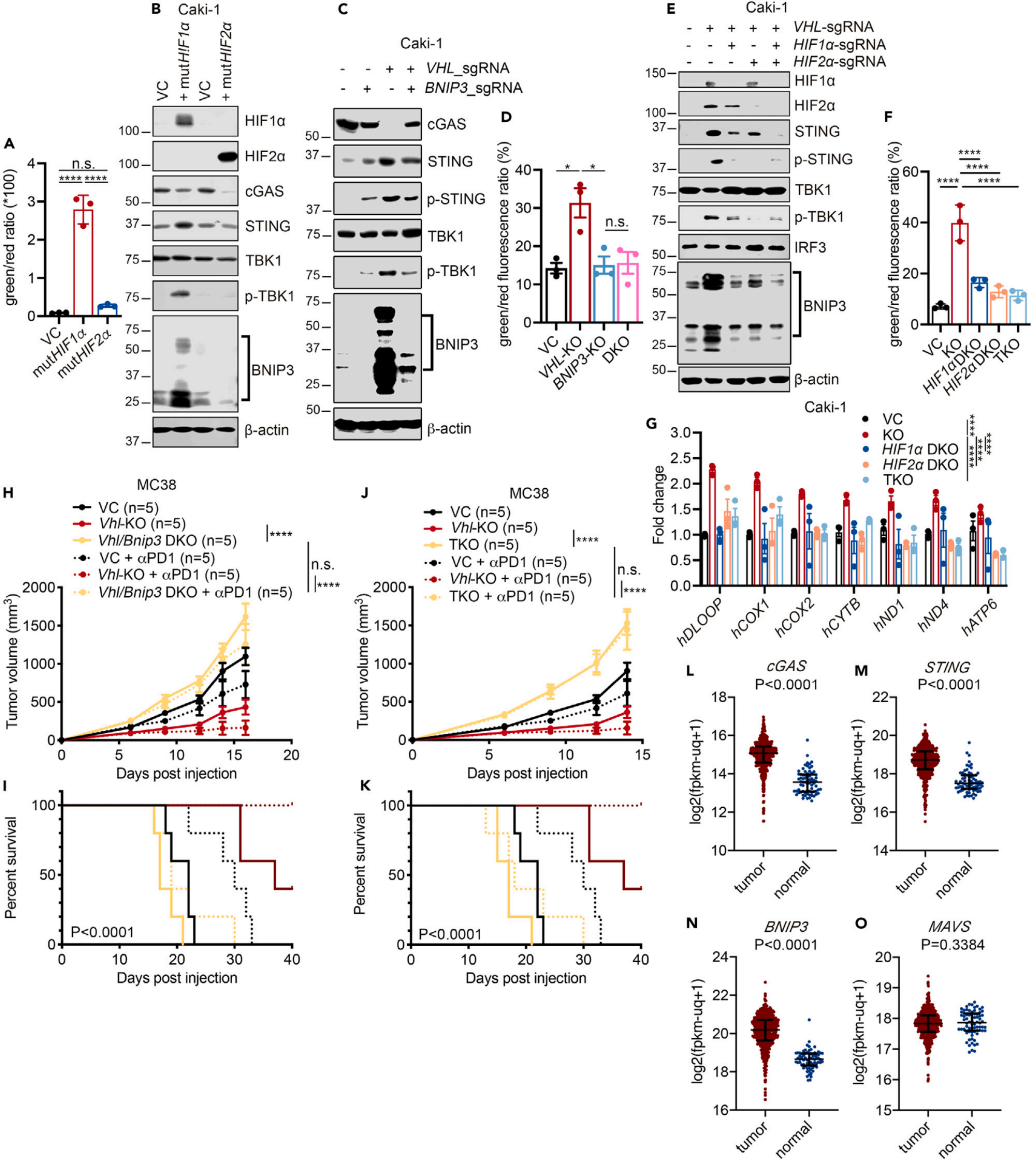
Elevated HIF1α and HIF2α levels are essential and sufficient for *VHL* deficiency-induced mitochondrial dysfunction and cGAS-STING activation (A) Quantitative JC-1-based MtMP measurements in Caki-1 cells expressing VC, degradation-resistant mut*HIF1α* (h*HIF1α*-p402A/p564A), or mut*HIF2α* (h*HIF2α*-p405A/p531A) by flow cytometry. Error bars represent mean ± SD. (B) Immunoblot analysis of cGAS-STING signaling proteins and BNIP3 in Caki-1 cells expressing exogenous VC, mut*HIF1α*, or mut*HIF2α* genes. (C) Immunoblot analysis of cGAS-STING signaling proteins in VC, *BNIP3*-KO, *VHL*-KO, and *VHL*/*BNIP3* DKO Caki-1 cells. (D) JC-1 assay for MtMP measurements in VC, *VHL*-KO, *BNIP3*-KO, and *VHL*/*BNIP3* DKO Caki-1 cells. Error bars represent mean ± SEM. (E) Immunoblot analysis of cGAS-STING signaling proteins and BNIP3 in VC, *VHL*-KO, *VHL*/*HIF1α* DKO, *VHL*/*HIF2α* DKO, and *VHL*/*HIF1α*/*HIF2α* TKO Caki-1 cells. (F) JC-1-based MtMP measurements in VC, *VHL*-KO, *VHL*/*HIF1α* DKO, *VHL*/*HIF2α* DKO, and *VHL*/*HIF1α*/*HIF2α* TKO Caki-1 cells by flow cytometry. Error bars represent mean ± SD. (G) Quantitative PCR measurements of cytosolic mtDNAs in VC, *VHL*-KO, *VHL*/*HIF1α* DKO, *VHL*/*HIF2α* DKO, and *VHL*/*HIF1α*/*HIF2α* TKO Caki-1 cells. (H and I) Tumor growth (G) and Kaplan-Meier analysis (H) of C57BL/6 mice subcutaneously inoculated with VC, *Vhl*-KO, or *Vhl/Bnip3* DKO MC38 cells and treated with isotype control or an αPD-1 antibody (100 μg per mouse). (J and K) Tumor growth (I) and Kaplan-Meier analysis (J) of C57BL/6 mice implanted with VC, *Vhl*-KO, or *Vhl/Hif1a/Hif2a* TKO MC38 cells and treated with isotype control or an αPD-1 antibody (100 μg per mouse). (G and I) Treatments were given on days 6, 9, and 12 post tumor cell inoculation. *n* = 5 per group. (L–O) Normalized mRNA expression levels of *cGAS* (K), *STING* (L), *BNIP3* (M), and *MAVS* (N) in tumor tissues and normal tissues from the TCGA_KIRC (ccRCC) cohort. Tumor tissues have higher levels of expressions with the exception of MAVS, which is not part of the cGAS-STING dsDNA-sensing pathway. Shown are results from three independent experiments. Error bars represent mean ± SEM. Two-way ANOVA. ∗*p* < 0.05; ∗∗*p* < 0.01; ∗∗∗*p* < 0.001; ∗∗∗∗*p* < 0.0001; n.s. not significant.

We next sought to understand the molecular mechanism of how *VHL* deficiency causes the reduction in MtMP. Because HIF1α and HIF2α are direct targets of VHL and are associated with hypoxia-induced mitochondrial dysfunction^2^^,^^3^^,^^14^^,^^54^^,^^55^ and *VHL*-deficient cells had elevated levels of HIF1α and HIF2α (Figures 4C and 4D), we decided to test whether forced expression of exogenous *HIF1α* and *HIF2α* could activate the cGAS-STING pathway. Therefore, we introduced into Caki-1 cells mutant versions of h*HIF1α* or h*HIF2α*, both of which are resistant to prolyl hydroxylase-induced hydroxylation and thus resistant to VHL-mediated degradation. Ectopic expression of mut*HIF1α* significantly enhanced monomer/aggregates ratio in JC-1-stained Caki-1 cells compared with control cells (Figures 6A and S6E), indicating a dramatic reduction in MtMP. In comparison, expression of mut*HIF2α* only moderately reduced MtMP (Figures 6A and S6E). Furthermore, expression of mut*HIF1α*, but not mut*HIF2α*, induced elevated expression of STING and activation of TBK1 (as indicated by detection of p-TBK1) in Caki-1 cells (Figure 6B), similar to those observed in *VHL*-KO cells (Figure 4D). Furthermore, similar to *VHL* KO, mut*HIF1α* also induced the activation of BNIP3 in Caki-1 cells (Figure 6B), consistent with it being a direct transcriptional target of HIF1α. To test whether BNIP3, that is upregulated by *VHL*-KO-mediated HIF1α accumulation, is required to promote cGAS-STING signaling activation, we further generated a mixed cell population of *BNIP3* knockdown and *VHL/BNIP3* double knockdown (DKD) using CRISPR-Cas9 system (Figures 6C and S6G). *VHL/BNIP3* DKD almost completely abrogated *VHL*-KO-induced activation of STING and TBK1 (Figures 6C and S6G). The MtMP was also restored with the depletion of BNIP3 (Figure 6D), indicating the change induced by *VHL* loss in MtMP is mediated by BNIP3. Therefore, elevated cGAS-STING signaling due to *VHL* loss is most likely regulated by the VHL-HIF1α-BNIP3 axis.

We further investigated whether HIF1α and HIF2α were essential for reducing MtMP and causing cytosolic mtDNA leakage in VHL-deficient cells. For this purpose, we generated *VHL/HIF1α DKO*, *VHL/HIF2α DKO,* and *VHL/HIF1α/HIF2α* triple-KO (TKO) Caki-1 cells, respectively (Figure 6E). Notably, KO of either *HIF1α* or *HIF2α* or both significantly reduced *VHL*-KO-induced activation of BNIP3 and cGAS-STING signaling (Figure 6E). This result was different from those in Figure 6A, where only forced expression of a stabilized mutHIF1α but not the stabilized mutHIF2α reduced MtMP. We suspect this discrepancy was caused by the different VHL background where the functions of HIF1α and HIF2α are examined. In Figures 6A and 6B, the VHL was WT while in Figures 6E and 6F, VHL was knocked out. In order to examine the role of VHL background on the functions of mutHIF1α and mutHIF2α in disrupting MtMP, we overexpressed the mutHIF1α and mutHIF2α in *VHL*-KO Caki-1 cells, respectively. Our results showed that *HIF1a* overexpression in *VHL*-KO cells further boosted the already activated BNIP3, similar to what is seen in cells with WT VHL (Figure S6F). On the other hand, different from *HIF2a* overexpression in *VHL*-WT cells, exogenous HIF2α in *VHL*-KO cells elevated BNIP3 activation and TBK1 activation compared to *VHL*-KO control cells (Figure S6F), suggesting differential abilities of HIF2α in *VHL*-WT and *VHL*-deficient cells.

Therefore, we conclude that, in *VHL-KO* cells, both HIF1α and HIF2α stabilization are required to activate cGAS-STING signaling. In agreement with this finding, *VHL-*KO-mediated reduction of MtMP was largely rescued in *VHL/HIF1α* DKO, *VHL/HIF2α* DKO, and *VHL/HIF1α/HIF2α* TKO cells, suggesting that both HIF1α and HIF2α are required for the *VHL* deficiency-induced mitochondrial membrane permeabilization (Figures 6F and S6H). Consistent with this finding, individual or combined *HIF1/2α* deletions in *VHL-KO* cells significantly reduced cytosolic mtDNA (Figure 6G), while changes of cytosolic nucDNA were insignificant (Figure S6I). Importantly, further KO of *Bnip3* and *Hif1α/2α* abrogated tumor growth delay observed in *Vhl-KO* MC38 tumors. In addition, loss of *Bnip3* or *Hif1α/Hif2α* in *Vhl*-deficient MC38 tumors also attenuated their sensitivity to ICB treatments significantly (Figures 6H–6K). Taken together, our results suggest that HIF1α and HIF2α are the critical effectors downstream of VHL responsible for reducing MtMP and increasing membrane permeability via BNIP3 to activate cGAS-STING signaling.

Many human tumors are resistant to immunotherapy because they downregulate STING signaling by various mechanisms,^56^^,^^57^ a fact that testifies to the importance of the cGAS-STING pathway in mediating tumor response to ICB therapy. In order to see if the cGAS-STING pathway is indeed upregulated in ccRCC as has been shown in our experiments, we analyzed the mRNA expression levels of *cGAS* and *STING* in a cohort of patients with ccRCC from the TCGA Pan-Cancer cohort. Our analysis indicated that both *cGAS* (Figure 6L) and *STING* (Figure 6M) were expressed at higher levels in tumor vs. adjacent normal tissues, thereby confirming the activation of the cGAS-STING pathway. Furthermore, *BNIP3* was similarly expressed at higher levels in tumor tissues (Figure 6N). Therefore, data from patients with ccRCC are consistent with the activation of the mtDNA-cGAS-STING pathway. In contrast, expression of mitochondrial antiviral signaling protein (MAVS), a vital component of the dsRNA-sensing pathway, showed no difference between tumor and adjacent normal tissues (Figure 6O). Taken together, our analysis of the TCGA human ccRCC data is consistent with the activation of the mtDNA-cGAS-STING pathway in these patients, the majority of whom have VHL deficiencies.

## Discussion

Our finding of *VHL* gene loss causing constitutive activation of the cGAS-STING signaling and increased intratumoral lymphocyte infiltration (Graphic Abstract) provides critical insights into how ccRCC with moderate TMB unexpectedly responds well to ICB therapy,^58^^,^^59^ despite the discrepancy that several published clinical studies did not show an improved response to ICB treatment in patients with VHL-mut ccRCC. We surmise that there could be several factors. One confounding factor might be that patients from most of the published studies also received different co-treatments. For example, in Javelin Renal 101,^60^ ImMotion150,^61^ and ImMotion 151^62^ RCC cohorts, patients receiving anti-PD-L1 treatment were also treated with VEGF inhibitors or anti-VEGF treatments concurrently. The other factor undermining the use of *VHL* genomic mutation as a biomarker for ICB therapy may be that *VHL* genomic mutations alone may not fully reflect the actual functional status of the *VHL* gene. In fact, even those patients with ccRCC with no *VHL* genomic mutations may have compromised VHL functions. For example, methylation-mediated *VHL* gene inactivation or loss of heterozygosity, which may occur in up to 98% of patients with ccRCC,^35^^,^^63^ may also disrupt VHL function. Therefore, even though we believe that compromised VHL function underlies the overall ccRCC responsiveness to ICB therapy, genomic mutations may not be a suitable biomarker for predicting individual ccRCC response to ICB therapy because of the prevalence of compromised VHL functions in ccRCC.

How do we reconcile the paradoxical roles of *VHL* loss, which promote tumor development in certain cancers, including ccRCC, and also predispose better response of tumors to ICB therapy? It may have to do with the dual roles of the cGAS-STING pathway, which acts downstream of VHL. Indeed, chronic cGAS-STING has been reported to promote tumor invasion and metastasis.^64^ In fact, the paradoxical roles of *VHL* mutations in promoting tumor development while making them susceptible to ICB therapy are not unique. Similar examples include mutations in the ataxia telangiectasia mutated gene and mismatch repair genes. Both are well-established tumor suppressor genes whose gene deficiency promotes tumorigenesis but makes them more susceptible to immunotherapy.^44^^,^^65^

Understanding the function of VHL in the immune response is complex. While its significance in tumor growth and angiogenesis is widely acknowledged, its effect on the immune system is just as significant and interrelated to these processes. Researchers are currently exploring how VHL and HIF signaling can influence immune responses. For instance, VHL and subsequent HIF1α stabilization can not only promote the expression of pro-inflammatory cytokines but also influence the fate and function of immune cells.^66^^,^^67^ However, VHL loss or mutations may also contribute to chronic inflammation and the development of inflammatory diseases.^68^^,^^69^^,^^70^ Therefore, further investigation regarding the intricate balance of VHL in inflammation is crucial for developing therapeutic strategies that leverage its benefits while mitigating its potential contributions to chronic inflammation and related diseases. Our efforts to uncover the precise mechanisms of VHL behind cancer immunotherapy could potentially lead to new therapeutic applications.

In addition, the constitutive cGAS-STING activation in *VHL*-deficient tumors can potentially create additional therapeutic opportunities beyond ICB immunotherapy. For example, *VHL*-deficient tumors may be more susceptible to chimeric antigen receptor T (CAR-T) therapy due to elevated levels of type I IFNs in the tumor microenvironment. Indeed, recent studies have shown that NKG2D CAR-engineered T cells can synergize with STING agonists in suppressing the growth of pancreatic tumors.^71^ By the same token, adoptively transferred tumor-infiltrating lymphocytes may have a better chance of penetrating the tumor mass and staying active due to a more hospitable TIME created by constitutive cGAS-STING activation.^72^^,^^73^ Another possibility is using STING agonists in combination with ICB therapy in RCC. The agonists may “supercharge” the constitutively high STING levels and create a more immune-stimulating tumor microenvironment that further enhances ICB therapy.^74^^,^^75^^,^^76^

In conclusion, our study provides insights into the *VHL* deficiency-induced cGAS-STING activation, providing a mechanistic underpinning of why tumors respond to ICB therapy. Finally, it also suggests potential treatment strategies for targeting *VHL*-deficient tumors based on constitutive cGAS-STING and type I IFN activation.

### Limitations of the study

Our conclusions are mostly drawn from experiments conducted in murine tumor models. Therefore, whether there is a similar role for VHL mutation in ccRCC ICB therapy still awaits further studies in human patients. Furthermore, human studies in ccRCC have to resolve confounding factors such as high VHL mutation rates in patients with ccRCC, co-mutations with VHL, and frequent combination treatments such as kinase inhibitors. The status of cGAS-STING activation in patients with ccRCC also needs to be confirmed in human patients with cancer.

## STAR★Methods

### Key resources table

**Table:** 

REAGENT or RESOURCE	SOURCE	IDENTIFIER
**Antibodies**		

Trustain FcX^TM^ anti-mouse CD16/32	BioLegend	Cat #101320; RRID:AB_1574975
FITC anti–mouse CD45	BioLegend	30-F11; Cat #103108; RRID:AB_312973
Pacific blue anti–mouse CD3	BioLegend	145-2c11; Cat #100334; RRID:AB_2028475
Alexa Fluor 647 anti–mouse CD4	BioLegend	GK1.5; Cat #100424; RRID:AB_389324
APC/Fire^TM^ 750 anti–mouse CD8a	BioLegend	53–6.7; Cat #100766; RRID:AB_2572113
phycoerythrin (PE) anti–mouse NK1.1	BioLegend	PK136; Cat #108707; RRID:AB_313394
APC anti–γ/δTCR	BioLegend	GL3; Cat #118116; RRID:AB_1731813
PE anti–mouse F4/80	BioLegend	BM8; Cat #123110; RRID:AB_893486
phycoerythrin (PE) anti–human/mouse Granzyme B	BioLegend	QA16A02; Cat #372207; RRID:AB_2687031
Alexa Fluor 647 anti–mouse IFN-γ	BioLegend	XMG1.2; Cat #505816; RRID:AB_493315
PE anti–mouse FOXP3	BioLegend	MF-14; Cat #126404; RRID:AB_1089117
PE anti-mouse H2-Kb/H2Db antibody	BioLegend	28-8-6, cat#114607; RRID:AB_313598
PE anti-mouse H-2K^b^/H2D^b^ antibody	BioLegend	Cat #114607; RRID:AB_313598
rat IgG2 isotype	BioXCell	Cat #BE0089; RRID:AB_1107769
rat anti-mouse αPD-1	BioXCell	Cat #BE0146; RRID:AB_10949053
mouse IgG1 isotype	BioXCell	Cat #EB0083; RRID:AB_1107784
anti-IFNAR1 antibody	BioXCell	Cat #BE0241; RRID:AB_2687723
anti-CD4 antibody	BioXCell	Cat #BE0003-1; RRID:AB_1107636
anti-CD8b antibody	BioXCell	Cat #BE0223; RRID:AB_2687706
anti-NK1.1 antibody	BioXCell	Cat #BE0036; RRID:AB_1107737
anti-cGAS	Cell Signaling Technology	D3080; cat# 31659; RRID:AB_2799008
anti-cGAS	Cell Signaling Technology	D1D3G; Cat #15102; RRID:AB_2732795
anti-STING	Cell Signaling Technology	2P2F; Cat# 13647; RRID:AB_2732796
anti-p-STING (Ser365)	Cell Signaling Technology	D8F4W; Cat #72971; RRID:AB_2799831
anti-p-STING (Ser366)	Cell Signaling Technology	D7C3S; Cat #19781; RRID:AB_2737062
anti-TBK1/Nak	Cell Signaling Technology	D1B4, Cat #3504; RRID:AB_2255663
anti–p-TBK1/p-Nak (Ser172)	Cell Signaling Technology	D52C2, Cat #5483; RRID:AB_10693472
anti-IRF-3	Cell Signaling Technology	D83B9; Cat #4302; RRID:AB_1904036
anti-*p*-IRF3 (Ser396)	Cell Signaling Technology	D601M; Cat #29047; RRID:AB_2773013
anti-HA-Tag	Cell Signaling Technology	C29F4; Cat #3724; RRID:AB_1549585
anti-HSP60	Cell Signaling Technology	D307; Cat #4870; RRID:AB_2295614
anti-HDAC1	Cell Signaling Technology	Cat #2062; RRID:AB_2118523
anti-BNIP3	Cell Signaling Technology	D7U1T; Cat #44060; RRID:AB_2799259
anti-MDA5	Cell Signaling Technology	D74E4, Cat#5321; RRID:AB_10694490
anti-dsDNA	Millipore Sigma	AC-30-10; Cat #CBL186; RRID:AB_11213573
anti-HIF-1 alpha	Novus Biologicals	H1alpha67; Cat #NB100-105; RRID:AB_10001154
anti-HIF-2 alpha/EPAS1	Novus Biologicals	Cat #NB100-122; RRID:AB_10002593
anti-GAPDH	Proteintech	cat #60004-1-Ig; RRID:AB_2107436
Anti-VHL	Santa Cruz Biotechnology	VHL40; Cat #sc-135657; RRID:AB_2215955
anti-actin	Thermo Fisher Scientific	ACTN05-C4; Cat #MA5-11869; RRID:AB_11004139
Alexa Fluor® 488 goat anti-mouse IgG	Thermo Fisher Scientific	Cat #A28175; RRID:AB_2536161
Alexa Fluor® 555 goat anti-rabbit IgG	Thermo Fisher Scientific	Cat #A27039; RRID:AB_2536100

**Bacterial and virus strains**		

lentiCRISPRv2	Addgene	#52961; RRID:Addgene_52961
psPAX2	Addgene	#12260; RRID:Addgene_12260
pMD2.G	Addgene	#12259; RRID:Addgene_12259
lentiCRISPRv2 *neo* vector	Addgene	#98292; RRID:Addgene_98292
px330-mcherry	Addgene	#98750; RRID:Addgene_98750
pSpCas9(BB)-2A-GFP(PX458)	Addgene	#48138; RRID:Addgene_48138
HA-VHL-pRC/CMV	Addgene	#19999; RRID:Addgene_19999
pcDNA3-HA-HIF1α(P402A/P564A)	Addgene	#18955; RRID:Addgene_18955
pcDNA3-HA-HIF2α(P405A/P531A)	Addgene	#18956; RRID:Addgene_18956
HA-tagged mouse VHL ORF Clone	Origene	Cat #MR201630
LentiORF pLEX vector	Thermo Scientific	Cat #OHS4735

**Biological samples**		

Xenograft MC38 tumors	This paper	

**Chemicals, peptides, and recombinant proteins**		

Trizol® Reagent	Ambion by life technologies	Cat #15596018
SDS	Bio-Rad	Cat #1610302
30% Acrylamide/Bis Soln, 37.5:1, 500 mL	Bio-Rad	Cat # 1610158
Resolving Gel Buffer, 1 L	Bio-Rad	Cat # 1610798
Stacking Gel Buffer 1L	Bio-Rad	Cat # 1610799
4x Laemmli Sample Buffer	Bio-Rad	Cat # 1610747
fixation buffer	BioLegend	Cat #420801
Ammonium persulphate	GE healthcare	Cat # 17-1311-01
RPMI-1640	Gibco	Cat #11875-119
Geneticin™ Selective Antibiotic (G418 Sulfate)	Gibco	Cat #10131035
DMEM medium	Gibco	Cat #11995073
McCoy’s 5A (Modified) Medium	Gibco	Cat # 16600082
Sodium pyruvate 100mM	Gibco	Cat #11360070
OPTI-MEM® I	Gibco	Cat # 11058021
HBSS	Gibco	Cat #14025092
Trypsin-EDTA (0.25%)	Gibco	Cat #25200114
Trypan Blue Solution, 0.4%	Gibco	Cat #15250061
penicillin-Streptomycin	Gibco	Cat #15140122)
FBS	Hyclone	Cat # SH30396.03
1× intracellular staining perm wash buffer	Invitrogen	Cat #00-8333-56
TURBO^TM^ DNase	Invitrogen	Cat #AM2238
SuperScript II Reverse Transcriptase	Invitrogen	Cat #18064014
Paraformaldehyde	J.T. Baker^TM^	Cat #S898-07
DNase I	Millipore Sigma	SKU #10104159001
digitonin	Millipore Sigma	SKU #D141-100MG
BsmBI	NEB	Cat #R0580
BbsI	NEB	Cat #R0539S
Phusion® High Fidelity DNA Polymerase	NEB	Cat #M0530S
T4 DNA ligase	NEB	Cat #0202L
T4 Polynucleotide Kinase	NEB	Cat #0201S
1× red blood cell lysis buffer	Roche	Cat #11814389001
Puromycin	Sigma	Cat #8833
Ampicillin	Sigma	Cat #A0166
D-(+)-GLUCOSE SOLUTION 45% IN H2O	Sigma	Cat #G8769-100ML
1× RIPA buffer	Sigma	Cat #R0278
1× protease inhibitors	Sigma	Cat #P8340
Collagenase type IV	Sigma	Cat #C5138
ethidium Bromide (EtBr)	Sigma	Cat #E1510
chloroform	Sigma	Cat #C2432
bovine serum albumin	Sigma	Cat #A3983
Triton X-100	Sigma	Cat #T8787
Lipofectamine^TM^ 2000	Thermo Fisher Scientific	Cat #11668019
PBS (10X), pH 7.4	Thermo Fisher Scientific	Cat # 70011044
SuperSignal^TM^ West Pico PLUS Chemiluminescent Substrate	Thermo Fisher Scientific	Cat #34580
LIVE/DEAD^TM^ fixable dead cell staining	Thermo Fisher Scientific	Cat #L23105
VECTASHIELD® Antifade Mounting Medium with DAPI	VECTOR LABORATORIES	Cat #H-1200-10

**Critical commercial assays**		

JC-1(tetraethylbenzimidazolylcarbocyanine iodide) Mitochondrial Membrane Potential Assay Kit	Abcam	Cat #113850
Universal Mycoplasma Detection Kit	ATCC	Cat #30-1012K
qPCRBIO SyGreen Blue Mix Hi-ROX	Genesee Scientific	Cat #17-506C
QIAGEN® DNeasy Blood & Tissue kit	QIAGEN	Cat # 69506
Gene JET Gel extraction kit	Thermo Fisher Scientific	Cat #K0692
DNA Clean & Concentrator-5 Kit	ZYMO RESEARCH	Cat #D4004
ZR Plasmid Miniprep Classic Kit	ZYMO RESEARCH	Cat #D4016

**Deposited data**		

Caki-1 RNAseq data	This paper	GEO: GSE196509
786-O RNAseq data (Published)	GEO Database	GEO: GSE108229

**Experimental models: Cell lines**		

HEK293T	ATCC	CRL3216™; RRID:CVCL_0063
Renca	ATCC	CRL2947™; RRID:CVCL_2174
B16F10	ATCC	CRL6475™; RRID:CVCL_A4CJ
Caki-1	ATCC	HTB-46™; RRID:CVCL_0234
786-O	ATCC	CRL1932™; RRID:CVCL_1051
MC38	Kerafast	ENH204-FP; RRID:CVCL_B288

**Experimental models: Organisms/strains**		

C57BL/6J	Jackson Laboratory	Strain #:000664; RRID:IMSR_JAX:000664
Balb/C	Jackson Laboratory	Strain #:000651; RRID:IMSR_JAX:000651

**Oligonucleotides**		

random hexamer primers	Invitrogen	Cat #SO142
sgRNA for CRISPR/Cas9 mediated gene knockout	IDT (Integrated DNA Technology)	Table S1
Primers for ectopic gene expression	IDT (Integrated DNA Technology)	Table S2
Primers for quantitative RT-PCR and quantitative PCR	IDT (Integrated DNA Technology)	Table S3

**Software and algorithms**		

ImmunoSEQ® mouse TCR-β CDR3 survey sequencing and ImmunoSEQ® Analyzer 3.0	Adaptive Technologies	NA
FACS Canto II Flow Cytometer	BD	NA
Astrios Sorter	Duke Cancer Institute flow cytometry core facility	NA
Prism	GraphPad	8.2.0
Illumina NovaSeq 6000	Illumina	NA
Leica TCS SP5 laser scanning confocal microscope	Leica	NA
Odyssey® Fc imaging system	LI-COR® Biosciences	NA
R	The R foundation	https://www.r-project.org/
Applied Biosystems® ViiATM 7 Real-Time PCR System with 384-well Block	Thermo Fisher Scientific	Cat #4453536

### Resource availability

#### Lead contact

Further information and requests for resources and reagents should be directed to and will be fulfilled by the lead contact (chuan.li@duke.edu).

#### Materials availability

This study did not generate new unique reagents. Plasmids and cell lines generated in this study are available upon request. All the other materials in this study are commercially available. Any additional analysis information for this work is available by request to the lead contact.

#### Data and code availability

Raw RNAseq and metadata are deposited in the Gene Expression Omnibus database and are publicly available as of the date of publication. The accession number is listed in the key resources table. The code for RNAseq analysis is provided in detail in the supplemental information. Any additional information required to reanalyze the data reported in this paper is available upon request. Source data for other figures will also be provided upon request from the lead contact.

### Experimental model and study participant details

#### Clinical samples and public datasets

All human data we obtained are publicly available. To compare the expression of *cGAS*, *STING*, *BNIP3*, and *MAVS* in RCC tumor versus normal tissues, we accessed three cohorts of studies from the open-access online tool cBioPortal (http://www.cbioportal.org).^77^^,^^78^ These studies include kidney renal clear cell carcinoma (KIRC), kidney renal papillary cell carcinoma (KIRP), and kidney chromophobe carcinoma (KICH) patients from the Cancer Genome Atlas (TCGA) Pan-Cancer studies.^79^^,^^80^^,^^81^

We used a published dataset (GSE108229) to perform gene ontology (GO) analysis. Top-ranked pathways were plotted with ‘ggplot2’ R package.

#### Cell lines and cell culture

We purchased the Renca mouse renal adenocarcinoma cells (ATCC CRL-2947), B16F10 mouse melanoma cells (ATCC CRL-6475), Caki-1 human clear cell carcinoma cells (ATCC HTB-46), and 786-O human renal cell adenocarcinoma (ATCC CRL-1932) from the Cell Culture Facility of Duke University School of Medicine. In addition, we obtained the MC38 mouse colon adenocarcinoma cells from Kerafast (Boston, MA). All cells were cultured and maintained following the manufacturer’s instructions. All cells undergo periodic mycoplasma testing using the Universal Mycoplasma Detection Kit from ATCC (Cat #30-1012K) to ensure they are mycoplasma-free.

#### *In vivo* tumor growth studies in mice

We purchased six-week-old C57BL/6J and Balb/C female mice from the Jackson Laboratory. Duke University Institutional Animal Use and Care Committee (IACUC) approved all mouse experiments in this study. For *in vivo* tumor growth experiments, we inoculated about 1∗10^6^ Renca cells, 1∗10^5^ B16F10 cells, and 5∗10^5^ MC38 cells (suspended in 50 μL 1× PBS) subcutaneously into the right flanks of syngeneic mice, respectively. The treatments were given via intraperitoneal injection according to the corresponding schedule. We measured tumor sizes by measuring the longest (length) and shortest (width) dimensions of the tumors every 2–3 days using a digital caliper and calculated tumor volumes using the following formula: (length) × (width)^2^/2. We euthanized mice when their volumes reached 2000 mm^3^.

In conducting immunotherapy in mice, we injected the antibodies intraperitoneally (i.p.), either with 100 μg of rat IgG2 isotype control (clone 2A3; Bio X Cell; Cat #BE0089), or 100μg of rat anti-mouse αPD-1 antibody in 150 μL 1×PBS per mouse on Day 5, 8, and 11 for Renca cells, on Day 7, 10, 13, and 16 for B16F10 cells, and on Day 6, 9, 12 for MC38 cells after tumor inoculation, respectively.

In experiments involving αIFNAR1 antibody treatments, we injected mice (i.p.) bearing MC38 tumors with 200 μg mouse IgG1 isotype control (clone MOPC-21; BioXCell; Cat #EB0083) or 200 μg mouse αIFNAR1 antibody (clone MAR1-5A3; BioXCell; Cat #BE0241) in 150 μL PBS per mouse on Day 5, 8, and 11, respectively.

In studies involving *in vivo* lymphocyte depletion, we injected 100 μg αCD4 antibody (Clone GK1.5; BioXCell; Cat #BE0003-1), or αCD8b antibody (Clone 53-5.8; BioXCell; Cat #BE0223), or 100 μg αNK1.1 antibody (Clone PK136; BioXCell; Cat #BE0036) into each MC38 tumor-bearing mouse on Day 1, 4, and 7 to deplete CD4^+^ T cells, CD8^+^ T cells, and NK cells, respectively. We also administered an equal amount of IgG isotype antibodies to control mice.

### Method details

#### CRISPR/Cas9-mediated gene knockout

We used the CRISPR/Cas9 system to generate gene-specific knockout cells. We designed single guided RNAs (sgRNAs) using a public domain web-based CRISPR sgRNA design tool CHOPCHOP (https://chopchop.cbu.uib.no). Table S1 lists individual sgRNA sequences targeting different genes. When generating Human *VHL*-KO, human *STING*-KO, human *HIF1α-*KO, human *HIF2α-*KO, mouse *Sting*-KO, and mouse *Mda5*-KO human and murine cancer cells, we used the lentiCRISPRv2 vector (Addgene #52961) following a published protocol from the Zhang lab.^82^ We digested LentiCRISPRv2 with BsmBI (NEB; Cat #R0580) and gel purified it using the Gene JET Gel extraction kit (Thermo Fisher Scientific; Cat #K0692). Oligos encoding sgRNA sequences were phosphorylated, annealed, and subsequently ligated into digested LentiCRISPRv2.

To produce sgRNA-encoding lentivirus vectors, we used HEK293T cells co-transfected with lentiviral constructs encoding the target sgRNAs and second-generation packaging plasmids psPAX2 (Addgene #12260) and pMD2.G (Addgene #12259) following the instruction from the Trono Lab (https://www.epfl.ch/labs/tronolab/laboratory-of-virology-and-genetics/lentivectors-toolbox/).

To generate clonal knockout cell lines, we infected target cells with sgRNA-encoding CRISPR/Cas9 lentivirus and cultured them in a complete cell growth medium, selected with puromycin (1 μg/ml for Caki-1) for 7–10 days. Cells were then collected to test the expression of target genes by immunoblot.

To generate clonal *VHL*-KO Caki-1 cells, cells were seeded in 96-well plates after a 7-day puromycin selection and screened for pure knockout clones verified by immunoblot analysis. To generate *VHL/STING* double knockout (DKO) cells, including *VHL/HIF1α* DKO, *VHL/HIF2α* DKO, or *VHL/HIF1α/HIF2α* triple knockout (TKO) Caki-1 cells, we cloned sgRNAs sequences encoding *HIF1α*, and/or *HIF2α* into digested lentiCRISPRv2 *neo* vector (Addgene #98292), respectively. We then infected *VHL*-KO Caki-1 cells with the sgRNA-encoding lentivirus and selected the cells with neomycin (2 mg/ml) for 10–14 days. Lentivirus prepared from lentiCRISPRv2 *neo* vector was used to generate control cells. We then detected protein levels of STING, HIF1α, and/or HIF2α knockdown (KD) by western blot. To generate *VHL/STING* DKO and *VHL/MDA5* DKO Caki-1 and MC38 cells, we infected *VHL-KO* Caki-1 cells with sgRNA-encoding lentivirus and cultured them in complete DMEM medium, and selected with puromycin (5 μg/ml for MC38 cells and 1 μg/ml for Caki-1 cells) for 7 days. We then seeded the cells in 96-well plates for single clone selection. We then verified the cells’ *VHL/STING* DKO and *VHL/MDA5* DKO status by immunoblot analysis.

To generate Vhl knockout in Renca, B16, or MC38 cells, we digested the px330-mcherry (Addgene #98750) and pSpCas9(BB)-2A-GFP(PX458) (Addgene #48138) with BbsI (NEB; Cat #R0539S), and then gel-purified, and ligated the vectors with annealed oligos encoding sgRNA_1 or sgRNA_2 targeting *Vhl*. We then transfected the vectors in the murine tumor cells with sgRNA-encoding constructs using Lipofectamine 2000 (Thermo Fisher Scientific; Cat #11668019). Cells transiently transfected with px330-mCherry and PX458 vectors alone were as controls (VC). We then cultured the cells using a complete DMEM medium [DMEM with 10% FBS and 100U/ml penicillin-Streptomycin (Gibco by Life Technologies; Cat #15140122)] for 6 days and subjected them to FACS sorting (Duke Cancer Institute flow cytometry core facility). We then seeded GFP^+^ mCherry^+^ cells in 96 well plates for single clone selection. We screened for VHL-KO single clones by immunoblot analysis. Table S1 shows the sgRNA primer sequences used for targeting various genes in this study.

#### Exogenous gene expression

We modified a commercially available LentiORF pLEX vector (Thermo Scientific; Cat #OHS4735) with EF-1α and used it to generate constructs for exogenous gene expression. Gene of interest was amplified by PCR using the Phusion High Fidelity DNA Polymerase. Specifically, we used HA-VHL-pRC/CMV (Addgene #19999) and HA-tagged mouse VHL ORF Clone (Origene; Cat #MR201630) as templates to amplify human and mouse VHL, respectively. In addition, we amplified mutant human HIF1/2α using the pcDNA3-HA-HIF1α(P402A/P564A) (Addgene #18955) plasmid and the pcDNA3-HA-HIF2α(P405A/P531A) plasmid (Addgene #18956). Modified pLEX vector was used for lentiviral production as controls. Table S2 lists the primer sequences for PCR reactions.

#### Immunoblot analysis

To prepare cellular lysates, we washed the cells quickly with ice-cold 1X PBS buffer twice and immediately lysed them in 1× RIPA buffer (Sigma; Cat #R0278) supplemented with 1× protease inhibitors (Sigma; Cat #P8340) on ice using a cell scraper. We then collected the lysates in a 1.5 mL tube and incubated them on ice for 10 min, then centrifuged them at 15,000 rpm for 15 min at 4°C. We then transferred the supernatants to a new 1.5 mL tube and boiled them for 5 min. We then loaded equal amounts of lysates into SDS-PAGE gels. After electrophoresis, we transferred the proteins to PVDF membranes, incubated them with primary antibodies overnight at 4°C, and then incubated them with HRP-conjugated secondary antibodies at room temperature for 1 h. Afterward, we incubated the membranes with the SuperSignal West Pico PLUS Chemiluminescent Substrate (Thermo Fisher Scientific; Cat #34580) and detected target signal strength with the Odyssey Fc imaging system (LI-COR Biosciences).

#### Flow cytometry

To analyze tumor-infiltrating lymphocytes, we implanted about 5∗10^5^ vector control or *Vhl*-KO, or *Vhl/Sting* DKO MC38 cells subcutaneously into the right flank of C57BL/6J mice. We euthanized tumor-bearing mice on day 15 post-inoculation. We removed and weighed the tumors and cut them into pieces as small as possible. We prepared 1× digestive enzyme solution with 0.2U/ml Collagenase type IV (Sigma; Cat #C5138) and 50 mg/mL DNase I (Millipore Sigma; SKU #10104159001) in HBSS. We then added the triple enzyme solution to the homogenates and incubated for 1 h at 37°C to ensure full digestion. We then passed the dissociated cells through a 70 μm cell strainer (BD Falcon; Cat #35–2350) and rinsed them with HBSS three times. The pellets were collected, lysed in 1× red blood cell lysis buffer (Roche; Cat #11814389001) on ice for 5 min, and rinsed with 1X wash buffer (2% FBS in 1X PBS). About 1∗10^6^ live cells were then blocked with TruStain FcX anti-mouse CD16/32 antibody (Clone 93; Biolegend; Cat #101320) on ice for 10 min, followed by LIVE/DEAD fixable dead cell staining (Thermo Fisher Scientific; Cat #L23105), and cell surface staining using perspective antibodies (see Antibodies and Reagents Section) on ice for 20 min. We then fixed the cells with fixation buffer (BioLegend; Cat #420801), permeabilized them with 1× intracellular staining perm wash buffer (Invitrogen; Cat #00-8333-56), and subjected them to intracellular staining using indicated antibodies (See Antibodies and Reagents Section). Samples were analyzed using BD FACS Canto II Flow Cytometer (Flow Cytometry Shared Facility, Duke University School of Medicine).

To evaluate the surface levels of MHC class I in VC and *Vhl*-KO MC38 cells, cells were detached by dissociation buffer (2% EDTA in 1× PBS), followed by blocking with TruStain FcX anti-mouse CD16/32 antibody (Clone 93; Biolegend; Cat #101320) on ice for 10 min. Cells were then stained with LIVE/DEAD fixable dead cell staining (Thermo Fisher Scientific; Cat #L23105) and PE anti-mouse H-2K^b^/H2D^b^ antibody (Clone 28-8-6; Biolegend; Cat #114607) on ice for 20 min in the dark, washed twice with 1× FACs buffer (2% FBS in 1×PBS with 2mM EDTA), and fixed with 1% PFA at room temperature for 15 min in the dark. Samples were washed twice, resuspended in 1× FACs buffer, and analyzed using BD FACS Canto II Flow Cytometer (Flow Cytometry Shared Facility, Duke University School of Medicine).

#### TCR sequencing

We implanted control and *Vhl*-KO MC38 cells into mice as described above. At 13 days post-inoculation, we euthanized the mice and collected tumor tissues, and extracted genomic DNA (gDNA) using QIAGEN DNeasy Blood & Tissue kit according to manufacturer’s instruction (QIAGEN; Cat # 69506). We sent about 3 μg gDNA (60 ng/μL in AE buffer) to Adaptive Technologies (Seattle, WA) for ImmunoSEQ mouse TCR-β CDR3 survey sequencing. Upon data acquisition, we analyzed them using ImmunoSEQ Analyzer 3.0, an Adaptive Biotechnologies online analysis platform.

#### Subcellular fractionation of cellular extracts

We followed a previously published procedure for subcellular fractionation of cellular extracts.^44^^,^^83^ Briefly, we seeded cells in 100 mm dishes two days before the experiment. On the day of the experiment, we divided 8∗10^6^ cells into two equal aliquots. We then suspended one aliquot in 500 μL of 50 mM NaOH, boiled the lysates for 30 min and used it to serve as total mtDNA control from whole cell lysate (WCL). Next, we resuspended the other aliquot in 500 μL of solution with 25 μg/mL of digitonin (Millipore Sigma; SKU #D141-100MG) in a buffer with 150mM of NaCl, 50mM HEPES at pH 7.4, and homogenized the cells by vigorous pipetting, and followed by a 10-min incubation period on ice to allow selective permeabilization of the plasma membrane. We then centrifuged the homogenates at 980 ×g three times for 3 min each at 4°C to pellet the intact cells. After the centrifugation, we rinsed the pellet with 1× PBS and used it as the pellet (Pel) fraction for immunoblot. Next, the supernatant was transferred to a new tube and centrifuged at 17,000 ×g for 10 min at 4°C to spin down the remaining cellular debris. The supernatant from this centrifugation was transferred to a new tube and used as the cytosolic (Cyto) fraction free of nuclear, mitochondrial, and endoplasmic reticulum contamination. We then boiled the WCL, Pel, and Cyto lysates at 95°C for 5 min and subjected them to western blot analysis to ensure the cytosolic preparation was free of contamination. Finally, we purified and concentrated both WCL_DNA and Cyto_DNA using DNA Clean & Concentrator-5 Kit (ZYMO RESEARCH; Cat #D4004) and used the DNA for quantitative PCR (qPCR) tests.

#### Depletion of cellular mtDNA

We depleted mtDNA using ethidium Bromide (EtBr) according to a previously published method.^84^^,^^85^ We treated VC and *VHL*-KO cells with 100 ng/mL of EtBr for 21 days with routine medium change every 2–3 days. We then validated the depletion of mtDNA by staining cells with anti-dsDNA and anti-HSP60 antibodies before we harvested the cells for immunoblot and qPCR.

#### Total RNA extraction

We plated about 6 ∗10^5^ VC and *VHL*-KO Caki-1 cells in 60 mm dishes two days before total RNA extraction. We then extracted total RNA using Trizol Reagent (Ambion by life technologies; Cat #15596018) according to a protocol from StarrLab (https://sites.google.com/a/umn.edu/starrlab/protocols/rna/rna-isolation-using-trizol). Briefly, we first rinsed the cells twice with ice-cold 1× PBS. We then added 1mL Trizol to lyse the cells. Next, we scraped the lysate off from the Petri dish and transferred it to a 1.5 mL Eppendorf tube, incubated the lysate at room temperature for 5 min, and added 200 μL chloroform (Sigma; Cat #C2432) with vigorous vortexing for 15 s. We then incubated the mixture at room temperature for 10 min and centrifuged it at 12,000 ×g for 15 min at 4°C. Next, we transferred the transparent top layer to a clean 1.5 mL tube, adding 500 μL isopropanol and incubating at room temperature for 10 min. The mixture was then centrifuged at 12,000 ×g for 10 min at 4°C to get a white pellet at the bottom. After removing the supernatant, the pellet was washed with 75% ethanol and centrifuged at 7,500 ×g for 5 min at 4°C. The pellet was then allowed to air dry and dissolved in 85 μL RNase-free H_2_O. Subsequently, we added 5 μL of TURBO DNase (Invitrogen by Thermo Fisher Scientific; Cat #AM2238) and 10 μL of 10× Turbo DNase buffer to degrade the remaining DNA at 37°C for 30 min. We then added 200 μL chloroform to stop the reaction. Afterward, we repeated the RNA extraction procedures as described above. Finally, we dissolved total RNA free of DNA contamination in RNase-free H_2_O and used it for quantitative PCR and bulk RNA sequencing analysis.

#### Quantitative RT-PCR (qRT-PCR) and quantitative PCR

To quantify RNA expression levels, we used the Trizol-extracted total RNA (described above) as the template for cDNA synthesis using random hexamer primers (Invitrogen by Thermo Fisher Scientific; Cat #SO142) and SuperScript II Reverse Transcriptase (Invitrogen Thermo Fisher Scientific; Cat #18064014) following the manufacturer’s instructions. Afterward, we performed qRT-PCR of the cDNA using qPCRBIO SyGreen Blue Mix Hi-ROX (Genesee Scientific; Cat #17-506C) and the Applied Biosystems ViiA 7 Real-Time PCR System with 384-well Block (Thermo Fisher Scientific; Cat #4453536). We used the comparative Ct (ΔΔCt) method to compare the relative changes in gene expression among different genes.

To quantify cytosolic DNA levels, we obtained cytosolic extracts as described above. We then conducted qPCR analysis using the cytosolic extracts as templates. To quantify cytosolic mitochondria DNA (mtDNA) and nuclear DNA (nucDNA) levels for any individual gene, we set the ratio between the cytosolic DNA level and the whole cell lysate as in the control cells as 1 and used it to obtain the relative levels of cytosolic DNA in other cells.

Table S3 lists the primers used for q-RT-PCR and qPCR analysis of different target genes.

#### JC-1 mitochondrial membrane potential assay

We measured mitochondrial membrane potential (MtMP) using the JC-1(tetraethylbenzimidazolylcarbocyanine iodide) mitochondrial membrane potential assay kit (Abcam; Cat #113850) following the manufacturer’s instruction. JC-1 is a lipophilic cationic carbocyanine dye that accumulates in mitochondria. In healthy mitochondria, the mitochondrial membrane is less permeable. The high mitochondrial membrane potential that results from the proton pump causes the aggregation of JC-1, which shows a red to orange color under fluorescence. However, damaged mitochondria are associated with highly permeable and depolarized mitochondrial membrane and reduced MtMP. Under such conditions, JC-1 predominantly forms monomers and produces a green fluorescence. We used FCCP (2-[2-[4-(trifluoromethoxy)phenyl]hydrazinylidene]-propanedinitrile) as a positive control for membrane depolarization as it is an uncoupler of oxidative phosphorylation in the mitochondria.^86^ We assessed the red fluorescence (excitation 535 nm)/emission 590 nm) and green fluorescence (excitation 475 nm/emission 530 nm) using immunofluorescence microscopy and flow cytometry. To quantify green/red fluorescence ratios in flow cytometry, the following formula was used: Ratio = [Red^+^Green^+^ (Q2, quadrant 2) + Red^−^Green^+^ (Q3)]/Red^+^Green^−^ (Q1).

#### Immunofluorescence microscopy

We seeded the cells in 35 mm glass-bottomed poly-D-lysine-coated dishes (MatTek Life Sciences; Cat #P35G-1.5-10-C) two days before experiments. We used 4% Paraformaldehyde (PFA) to fix cells at room temperature for 15 min and permeabilized the cells using 0.5% Triton X-100 in PBS at room temperature for 10 min. We then washed the cells three times with PBS and blocked them with 5% bovine serum albumin (BSA; Sigma; Cat #A3983) at room temperature for 1 h. Next, we added primary antibodies and incubated the cells at 4°C overnight, followed by adding fluorophore-conjugated secondary antibodies after washing with PBS three times. Next, we incubated the cells at room temperature for 1 h in the dark and washed them three times. Finally, we added VECTASHIELD Antifade Mounting Medium with DAPI (VECTOR LABORATORIES; Cat #H-1200-10) to the glass bottom of the dish before analysis. We took fluorescence images using the Leica TCS SP5 laser scanning confocal microscope in the Light Microscopy Core Facility of Duke University School of Medicine.

#### Bulk RNA sequencing

To perform genome-wide transcriptome analysis of VC and *VHL*-KO Caki-1 cells, we prepared total RNAs from the cells using Trizol Reagent as described above. We then submitted our RNA samples to the Duke Center for Genomic and Computational Biology for sequencing, which QC’ed the samples and prepared cDNA libraries for analysis using Illumina NovaSeq 6000. We processed the RNA-seq data using the TrimGalore toolkit, which employs Cutadapt to trim low-quality bases and Illumina sequencing adapters from the 3′ end of the reads.^87^^,^^88^ Only reads that were 20nt or longer after trimming were kept for further analysis. Reads were mapped to the GRCh38.p13 of the human genome and transcriptome using the STAR RNA-seq alignment tool.^89^^,^^90^ Reads were kept for subsequent analysis if they mapped to a single genomic location using the SAMtools.^91^ Gene counts were compiled using the HTSeq tool.^92^ Only genes that had at least 10 reads in any given library were used in subsequent analysis. Normalization and differential expression were carried out using the DESeq2 Bioconductor package with the R statistical programming environment. Software for gene set enrichment analysis (GSEA; version 4.1.0) was used to identify differentially regulated pathways. The source RNA-seq data are deposited in the NCBI’s Gene Expression Omnibus (GEO) database.

#### Analysis of published RNAseq data

To assess enriched pathways from VHL overexpression in 786-O cells, a previously published dataset (GSE108229) was used to perform gene ontology (GO) analysis.^93^ Top-ranked pathways were plotted with ‘ggplot2’ R package. Scripts are available in the supplemental information.

### Quantification and statistical analysis

Statistical analysis was conducted using GraphPad Prism 8.2.0 software. Two-sided Student’s t test was used for comparing two experimental groups. One-way ANOVA was applied to compare gene expression levels among multiple groups. two-way ANOVA was applied to compare *in vivo* tumor growth rates within two or more experimental groups. mtDNAs and nucDNAs levels in VC, *VHL*-KO, *VHL/HIF1α* DKO, *VHL/HIF2α* DKO, and *VHL/HIF1α/HIF2α* TKO Caki-1 cells were also analyzed using two-way ANOVA. Log rank (Mantel-Cox) test was used for mouse and human patient survival analysis. ∗*p* < 0.05 was considered statistically significant.

## Supplemental information

Document S1. Figures S1‒S6 and Tables S1–S3

Data S1. Pathway analysis of published RNA-seq data using Reactome

Data S2. Script for plotting pathways using the R program
