# Supplementary material for: VHL loss enhances antitumor immunity by activating the anti-viral DNA-sensing pathway
Source: iScience. 2024 Jun 15;27(7):110285. doi: 10.1016/j.isci.2024.110285 (PMC11267025; doi:10.1016/j.isci.2024.110285)
Supplement: Document S1. Figures S1‒S6 and Tables S1–S3 [file mmc1.pdf]

iScience, Volume *27*

Supplemental information

VHL loss enhances antitumor

immunity by activating

the anti-viral DNA-sensing pathway

Meng Jiao, Mengjie Hu, Dong Pan, Xinjian Liu, Xuhui Bao, Jonathan Kim, Fang Li, and Chuan-Yuan Li

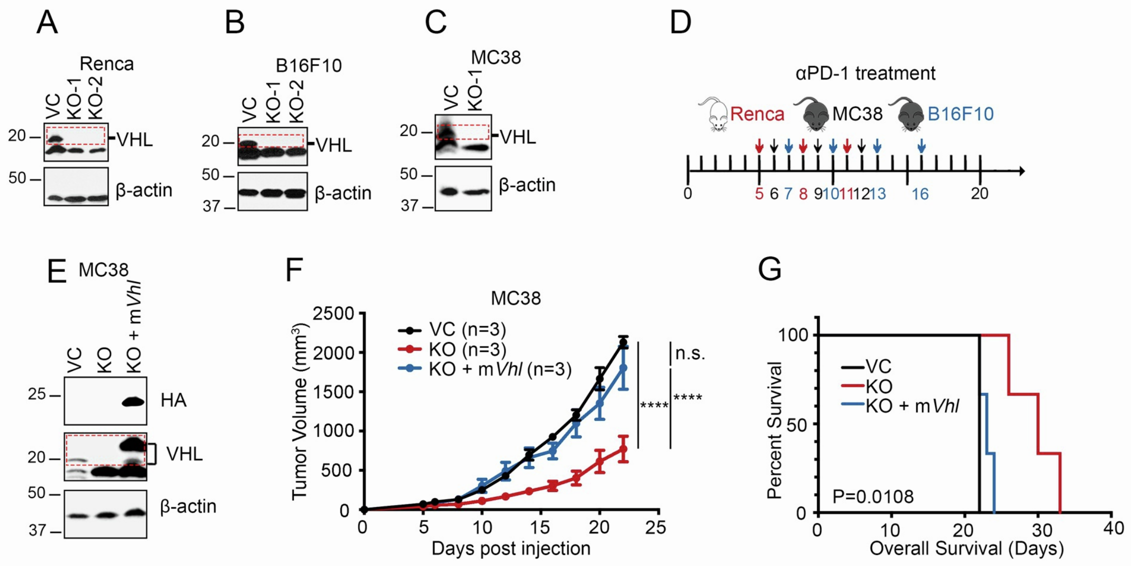

Figure S1. Additional data on the role of *Vhl* gene loss in tumor growth, related to Figure 1. (A-C) Immunoblot showing genomic knockout of *Vhl* in Renca (A), B16F10 (B), and MC38 (C) cells. **(D)** Scheduling of for αPD-1 antibody treatments in mice bearing Renca, B16F10, and MC38 tumors. **(E)** Immunoblot verification of the re-expression of wild-type mouse *Vhl (mVhl)* in *Vhl*-KO MC38 cells. **(F-G)** Tumor growth (F) and Kaplan-Meier survival curves (G) of C57BL/6J mice subcutaneously implanted with $5x10^5$ VC, *Vhl*-KO, and *Vhl*-KO MC38 cells re-expressing m*Vhl* (n=3). Error bars represent mean ± SEM. *P<0.05; **P<0.01; ***P<0.001; ****P<0.0001; n.s. not significant; two-way ANOVA.

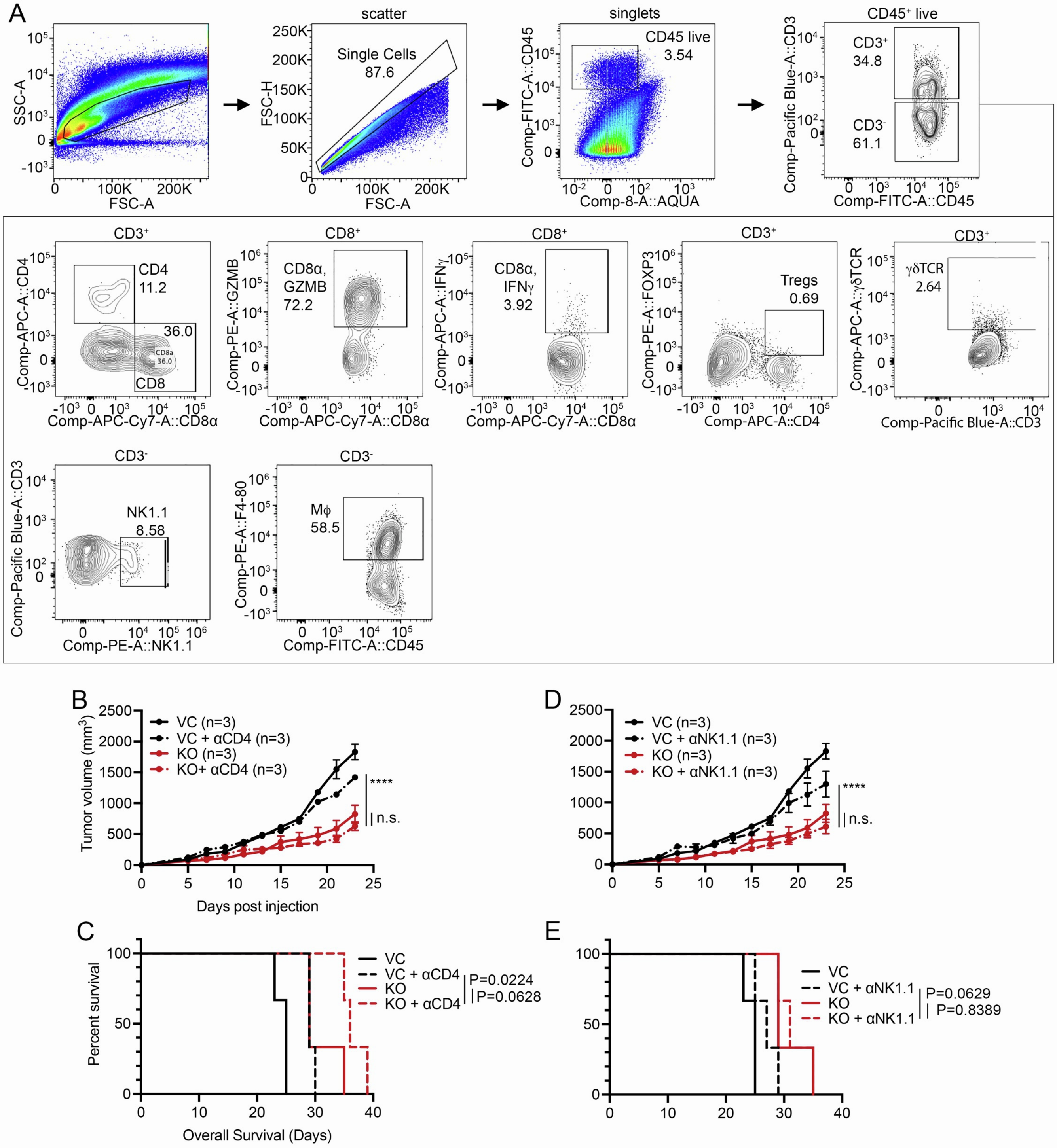

Figure S2. Additional data on the involvement of immune effector cells in *Vhl* loss mediated tumor growth delay, related to Figure 2. (A) Gating strategy of TIL analysis by flow cytometry. **(B-C)** Tumor growth (B) and Kaplan-Meier survival curves (C) of C57BL/6J mice bearing VC and *Vhl*-KO MC38 tumors in mice injected with isotype or αCD4 antibodies (n=3). **(D-E)** Tumor growth (D) and Kaplan-Meier survival curves (E) of C57BL/6J mice bearing VC and *Vhl*-KO MC38 tumor injected with isotype or αNK1.1 antibodies (n=3). In B and D, error bars represent mean $\pm$ SEM. *P<0.05; **P<0.01; ***P<0.001; ****P<0.0001; n.s. not significant; two-way ANOVA. In C and E, p values calculated by using the log-rank test.

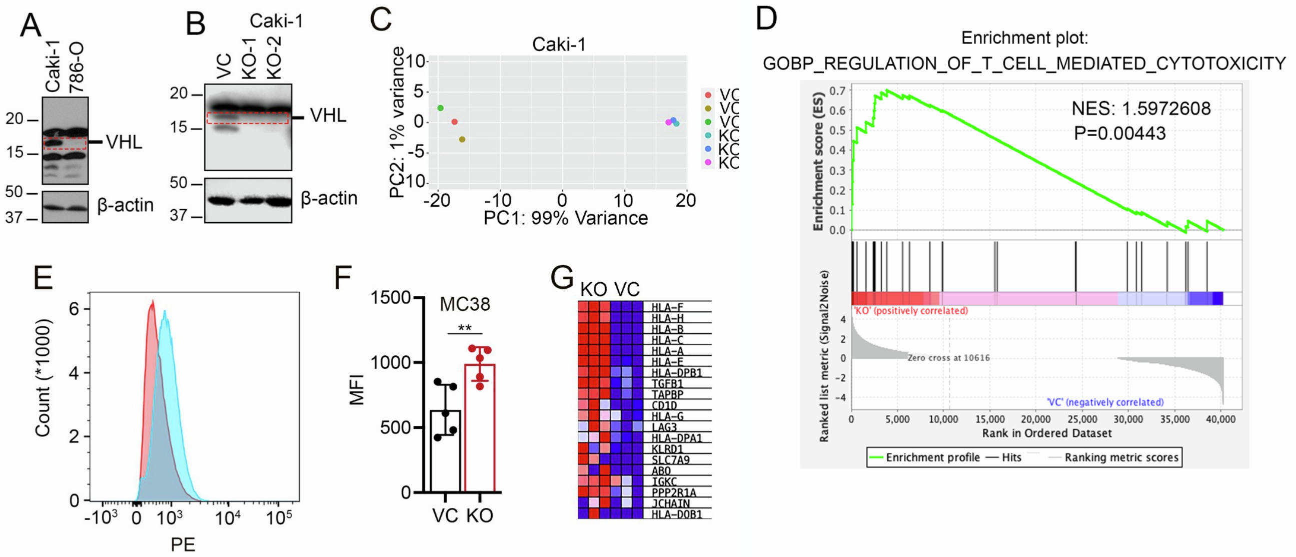

Figure S3. Supporting data of control and *VHL*-KO Caki-1 cells and comparison of surface MHC-I expression in Caki-1 cells, related to Figure 3. **(A)** Immunoblots of *VHL* expression in Caki-1 and 786-O cells. **(B)** Immunoblot analysis verifying the knockout of *VHL* in Caki-1 cells. **(C)** PCA analysis of RNA sequencing data generated from control and *VHL*-KO Caki-1 cells. **(D)** GSEA analysis indicating the enrichment of regulation of T cell mediated cytotoxicity in *VHL*-KO Caki-1 cells. (**E-F)** Flow cytometry analysis of the levels of H2K^b/H2D^b in VC and *Vhl*-KO MC38 cells. Data in F were from five independent experiments. **(G)** Heatmap of top-20 differently expressed genes involved in antigen binding in *VHL*-KO and control Caki-1 cells. Error bars represent mean ± SD. *P<0.05; **P<0.01; ***P<0.001; ****P<0.0001; n.s. not significant.

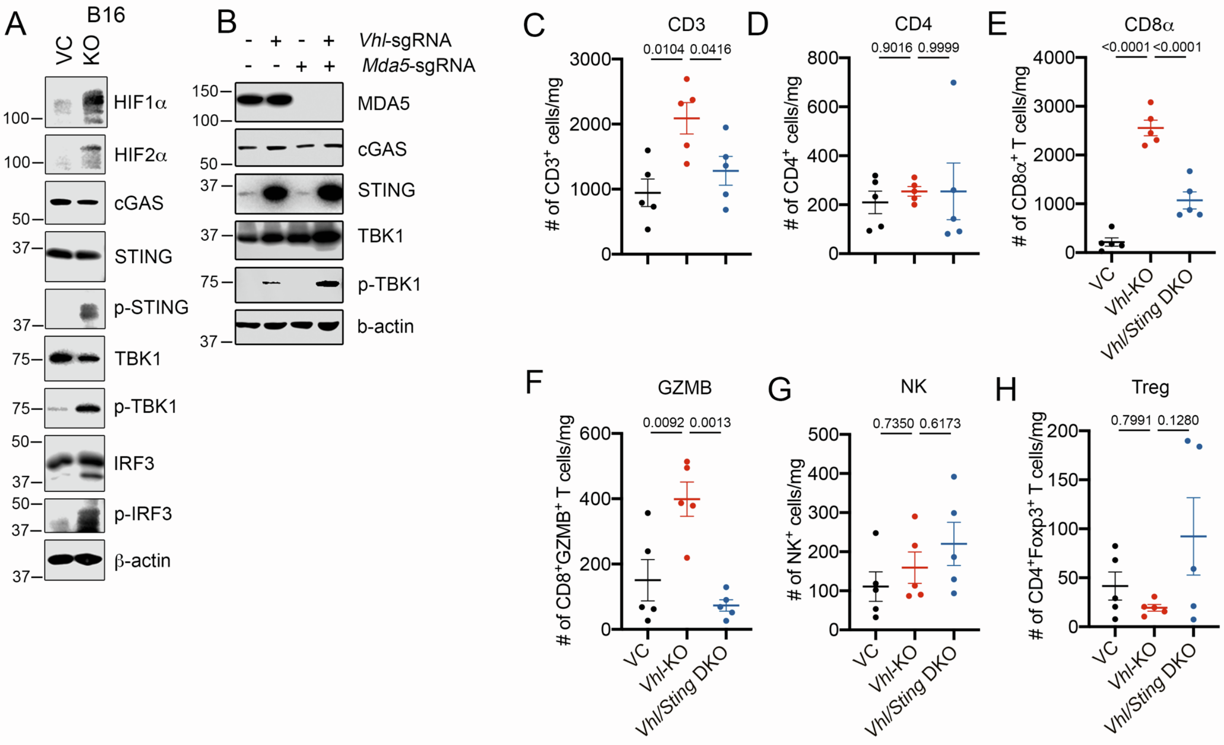

Figure S4. The importance of cGAS-STING mediated DNA sensing but not MDA5-mediated RNA sensing pathway in *Vhl*-KO induced anti-tumor immune responses, related to Figure 4. (A) Upregulation of cGAS-STING signaling and its downstream effectors in B16F10 cells with *Vhl* gene loss. **(B)** Immunoblot analysis of cGAS-STING and type I interferon signaling pathway in VC, *Vhl*-KO, *Mda5*-KO, and *Vhl*/*Mda5* DKO MC38 cells. **(C-H)** Tumor infiltrated lymphocytes from VC, *Vhl*-KO, and *Vhl/Sting* DKO MC38 tumors by flow cytometry (n=5 per group). Error bars represents mean ± SEM.

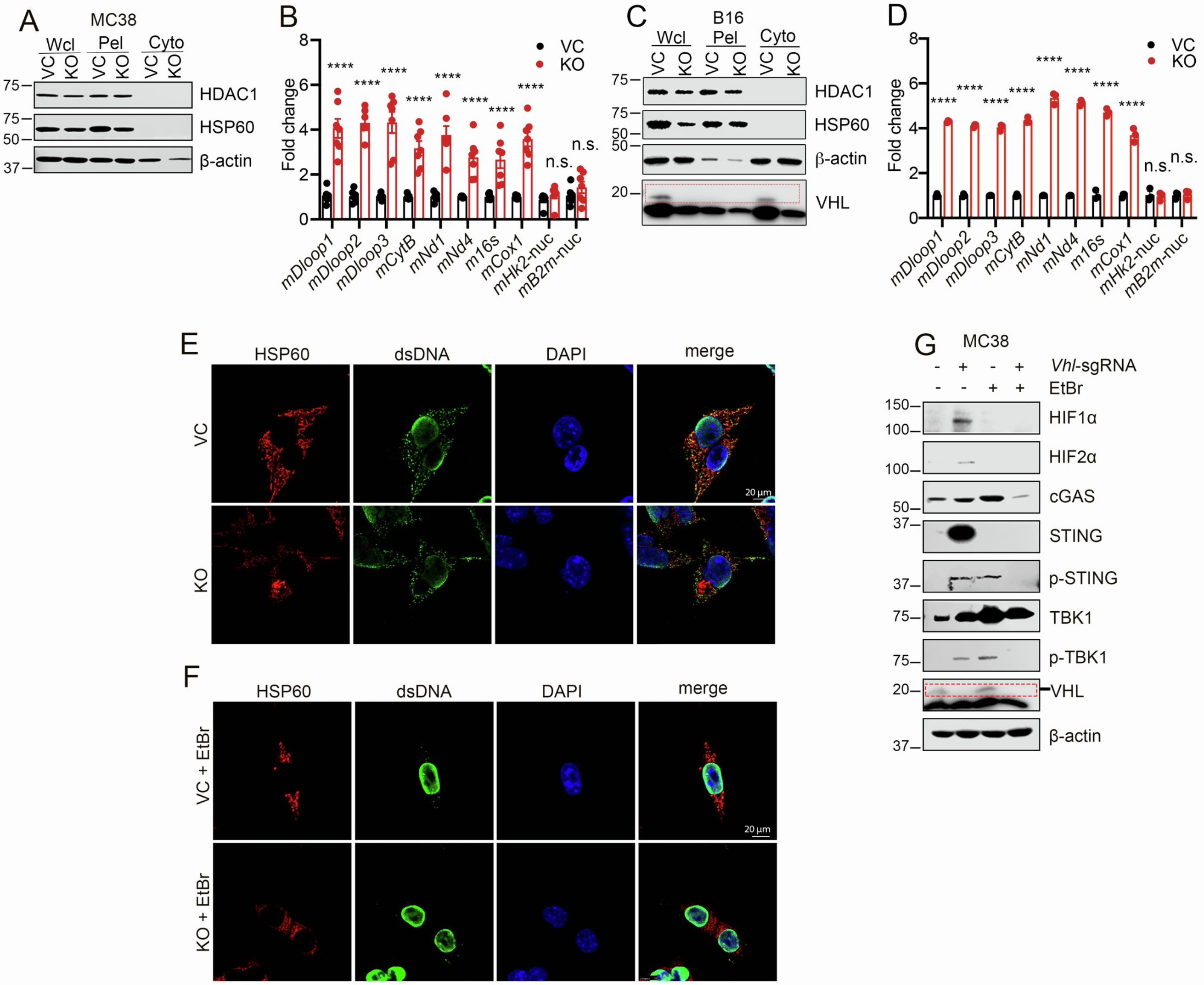

Figure S5. Additional data on *Vhl* loss induced mtDNA leakage, related to Figure 5. (A-D) Comparison of cytosolic mtDNA levels in VC and *Vhl*-KO cells. (A, C) Immunoblot analysis validating the purity of cytosolic extracts from VC and *Vhl*-KO MC38 (A) and B16F10 (C) cells. (B, D) Quantitative PCR analysis of cytosolic mtDNAs in VC and *Vhl*-KO MC38 (B) and B16F10 (D) cells. Results from three independent experiments. **(E)** Immunofluorescent microscopy of VC and *VHL*-KO MC38 cells stained with anti-HSP60 and anti-dsDNA antibodies. Scale bar, 20μM. **(F)** Immunofluorescent microscopy verifying mtDNA depletion in VC and *Vhl*-KO MC38 cells treated with 100 ng/ml EtBr. Scale bar, 20μM. **(G)** Immunoblot analysis of cGAS-SITNG signaling in VC and *Vhl*-KO MC38 cells treated with vehicles or 100 ng/ml EtBr. Error bars represent mean ± SEM. *P<0.05; **P<0.01; ***P<0.001; ****P<0.0001; n.s. not significant.

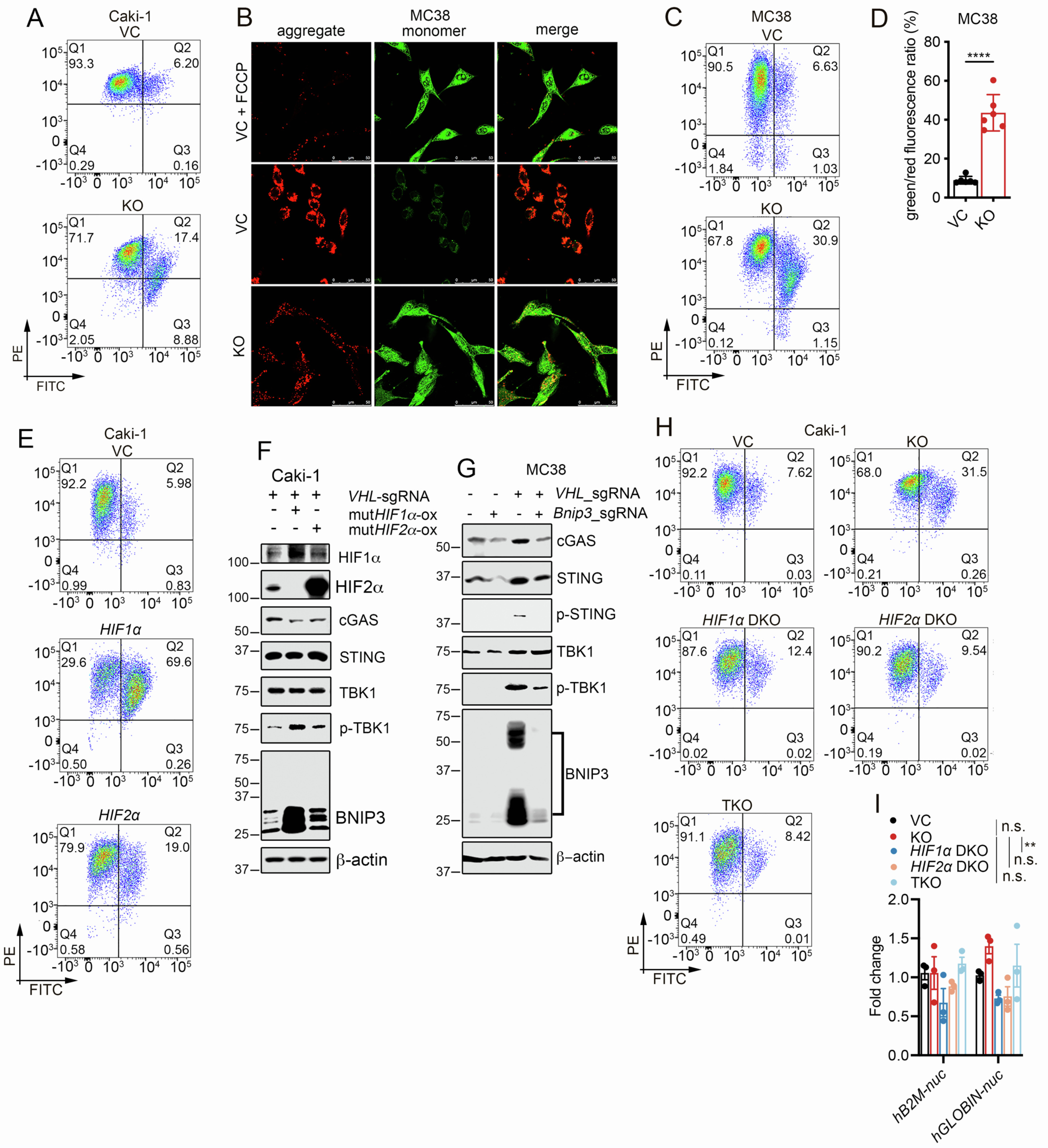

Figure S6. Additional data demonstrating that HIF1α and HIF2α are functionally required for the *VHL* deficiency-induced mitochondrial membrane permeabilization, related to Figure 6. (A) Flow cytometry of JC-1 staining of VC and *VHL*-KO Caki-1 cells. **(B-D)** JC1-enabled mitochondrial membrane potential (MtMP) assay for VC and *Vhl*-KO MC38 cells as detected by confocal microscopy (B) and flow cytometry (C-D). Scale bar, 50μM. Error bars represent mean ± SD. **(E)** Flow cytometry analysis JC-1 staining of VC, degradation-resistant mut*HIF1α* (h*HIF1α*-p402A/p564A), and mut*HIF2α* (h*HIF2α*-p405A/p531A) expressing Caki-1 cells. **(F)** Immunoblot analysis to assess cGAS-STING signaling in *VHL*-KO Caki-1 cells. The cells were transduced to express a vector control, mutHIF1α (hHIF1α-p402A/p564A), or mutHIF2α (hHIF2α-p405A/p531A), respectively. **(G)** Immunoblot analysis of cGAS-STING signaling proteins in VC, *Bnip3*-KO, *Vhl*-KO, and *Vhl*/*Bnip3* DKO MC38 cells. **(H)** Flow cytometry analysis of JC-1 staining in VC, *VHL*-KO, *VHL*/*HIF1α* DKO, *VHL*/*HIF2α* DKO, and *VHL*/*HIF1α*/*HIF2α* TKO Caki-1 cells. **(I)** Quantitative PCR analysis of cytosolic nucDNAs in VC, *VHL*-KO, *VHL*/*HIF1α* DKO, *VHL*/*HIF2α* DKO, and *VHL*/*HIF1α*/*HIF2α* TKO Caki-1 cells. Data from three independent experiments. Error bars represent mean ± SEM. Two-way ANOVA. *P<0.05; **P<0.01; ***P<0.001; ****P<0.0001; n.s. not significant.

Table S1: sgRNAs for CRISPR/Cas9 mediated gene knockout, related to STAR methods.

Target gene	SgRNA #	Sequences 5' – 3'
Mouse *Vhl*	sgRNA_1	CCGATCTTACCACCGGGCAC
Mouse *Vhl*	sgRNA_2	ACAAAGGCAGCACGACGCGC
Human *VHL*	sgRNA_1	CCCGTATGGCTCAACTTCGA
Human *VHL*	sgRNA_2	CATACGGGCAGCACGACGCG
Mouse *Sting*	sgRNA_1	CTTCTCGCTACAACACATGA
Human *STING*	sgRNA_1	CATTACAACAACCTGCTACG
Human *STING*	sgRNA_2	GCTGGGACTGCTGTTAAACG
Mouse *Mda5*	sgRNA_1	GGCAGGGATTCAGGCACCAT
Human *HIF1α*	sgRNA_1	AAGTGTACCCTAACTAGCCG
Human *HIF1α*	sgRNA_2	AGATGCGAACTCACATTATG
Human *HIF2α*	sgRNA_1	ACGAATCTCCTCATGGTCGC
Human *HIF2α*	sgRNA_2	GCTGATTGCCAGTCGCATGA
Human *BNIP3*	sgRNA_1	CGCCCATTGGCCGCGACTTG
Human *BNIP3*	sgRNA_2	TTCTGCGACATGGCGCCAGA
Mouse *Bnip3*	sgRNA_1	CGAGCCACCATGTCGCAGAG
Mouse *Bnip3*	sgRNA_2	GCGACATGGTGGCTCGGCAA

Table S2: Primers for ectopic gene expression, related to STAR methods.

Target gene for ectopic expression	Sequence 5' – 3'
Mouse *Vhl* forward primer	CCGACTCTACTAGAGGATCCACTAGTGCCACCATGCCCCGGAAGGCAGCCAG
Mouse *Vhl* reverse primer	CAGGAACATCATACGGATAAGCGGCCGCAGGCTCCTCTTCCAGGTGCT
Human *VHL* forward primer	CCGACTCTACTAGAGGATCCACTAGTGCCACCATGTACCCCTACGACGTGCC
Human *VHL* reverse primer	CAGGAACATCATACGGATAAGCGGCCGCTCAATCTCCCATCCGTTGAT
Human *HIF1α*-p402A/p564A forward primer	CCGACTCTACTAGAGGATCCACTAGTGCCACCATGTACCCCTACGACGTGCC
Human *HIF1α*-p402A/p564A reverse primer	CAGGAACATCATACGGATAAGCGGCCGCTCAGTTAACTTGATCCAAAG
Human *HIF2α*-p405A/p531A forward primer	CCGACTCTACTAGAGGATCCACTAGTGCCACCATGACAGCTGACAAGGAGAA
Human *HIF2α*-p405A/p531A reverse primer	CAGGAACATCATACGGATAAGCGGCCGCGGTGGCCTGGTCCAGGGCTC

Table S3: Primers for quantitative RT-PCR and quantitative PCR, related to STAR methods.

Target gene	Primer	Sequences 5' – 3'
Human mtDNA *DLOOP*	Forward primer	AGCACATTACAGTCAAATCCCTTCTC
	Reverse primer	CACGGAGGATGGTGGTCAAG
Human mtDNA *COX1*	Forward primer	ATATTTCACCTCCGCTACCA
	Reverse primer	TCAGCTAAATACTTTGACGCC
Human mtDNA *COX2*	Forward primer	ACGCATCCTTTACATAACAGAC
	Reverse primer	GCCAATTGATTTGATGGTAAGG
Human mtDNA *CYTB*	Forward primer	ATCACTTTATTGACTCCTAGCC
	Reverse primer	TGGTTGTCCTCCGATTCAG
Human mtDNA *ND1*	Forward primer	CCCTAAAACCCGCCACATCT
	Reverse primer	GAGCGATGGTGAGAGCTAAGGT
Human mtDNA *ND4*	Forward primer	CCCTTCCTTGTACTATCCCT
	Reverse primer	TTTGTCGTAGGCAGATGGAG
Human mtDNA *ATP6*	Forward primer	TCCCTCTACACTTATCATCTTCAC
	Reverse primer	GACAGCGATTTCTAGGATAGTC
Human nucDNA *B2M*	Forward primer	GTGCCTGATATAGCTTGACACCAA
	Reverse primer	TCGGGAAAAGACACATTAATATTGCCA
Human nucDNA *GLOBIN*	Forward primer	CTATGGGACGCTTGATGT
	Reverse primer	GCAATCATTCGTCTGTTT
mouse mtDNA *Dloop1*	Forward primer	AATCTACCATCCTCCGTGAAACC
	Reverse primer	TCAGTTTAGCTACCCCCAAGTTTAA
mouse mtDNA *Dloop2*	Forward primer	CCCTTCCCCATTTGGTCT
	Reverse primer	TGGTTTCACGGAGGATGG
mouse mtDNA *Dloop3*	Forward primer	TCCTCCGTGAAACCAACAA
	Reverse primer	AGCGAGAAGAGGGGCATT
mouse mtDNA *CytB*	Forward primer	GCTTTCCACTTCATCTTACCATTTA
	Reverse primer	TGTTGGGTTGTTTGATCCTG
mouse mtDNA *16S*	Forward primer	CACTGCCTGCCCAGTGA
	Reverse primer	ATACCGCGGCCGTTAAA
mouse mtDNA *Nd1*	Forward primer	CTAGCAGAAACAAACCGGGC
	Reverse primer	CCGGCTGCGTATTCTACGTT
mouse mtDNA *Nd4*	Forward primer	AACGGATCCACAGCCGTA
	Reverse primer	AGTCCTCGGGCCATGATT
mouse mtDNA *Cox1*	Forward primer	GCCCCAGATATAGCATTCCC
	Reverse primer	GTTCATCCTGTTCCTGCTCC

mouse nucDNA *Hk2*	Forward primer	GCCAGCCTCTCCTGATTTTAGTGT
	Reverse primer	GGGAACACAAAAGACCTCTTCTGG
mouse nucDNA *B2m*	Forward primer	ATGGGAAGCCGAACATACTG
	Reverse primer	CAGTCTCAGTGGGGGTGAAT
mouse $Ifn\alpha$	Forward primer	GGATGTGACCTTCCTCAGACTC
	Reverse primer	ACCTTCTCCTGCGGGAATCCAA
mouse $Ifn\beta$	Forward primer	CTGGCTTCCATCATGAACAA
	Reverse primer	AGAGGGCTGTGGTGGAGAA
mouse *β–actin*	Forward primer	GAAATCGTGCGTGACATCAAA
	Reverse primer	TGTAGTTTCATGGATGCCACA
