# Supplementary material for: VHL loss enhances antitumor immunity by activating the anti-viral DNA-sensing pathway
Source: iScience. 2024 Jun 15;27(7):110285. doi: 10.1016/j.isci.2024.110285 (PMC11267025; doi:10.1016/j.isci.2024.110285)
Supplement: Data S2. Script for plotting pathways using the R program [file mmc3.pdf]

```
rm(list=ls())
setwd("~/Desktop/Liao 2018 786 VHL ox RNAseq/")
library(ggplot2)
library(dplyr)
library(stringr)
library(scales)
df <- read.csv("Data S1_Pathway analysis of published RNAseq data using
Reactome")
colnames(df)
df <- df[1:10,] # select the top pathways to plot
# order pathways based on FDR
df$Pathway.name <- factor(df$Pathway.name, levels =
df$Pathway.name[order(df$Entities.FDR, decreasing = T)])
p <- ggplot(df, aes(y=Pathway.name, x=-log10(Entities.FDR), colour=
Entities.ratio, size = X.Entities.found)) + geom_point()
p +  labs(x="-log10(FDR)", y = "pathways", colour="Entity ratio",
size="Entity count") + scale_color_gradient(low = "red", high = "black") +
theme(text=element_text(size=16))




###
df3 <- df[1:10,c(2,3,5,7)]
df3 <- mutate(df3, "-log10(FDR)" = -log10(df3$Entities.FDR))
df3$Pathway.name <- factor(df3$Pathway.name, levels =
df3$Pathway.name[order(df3$Entities.FDR, decreasing = T)])

p <- ggplot(df3, aes(y=X.Entities.found, x=Pathway.name, colour=`-
log10(FDR)`, size = Entities.ratio)) + geom_point()
p + coord_flip() + labs(x="", y = "entities", colour="-log10(FDR)",
size="%entities") +
  scale_x_discrete(labels = function(x) str_wrap(x, width = 50)) + ylim(0,
60) + theme(text = element_text(size=15))

p <- ggplot(df3, aes(y=X.Entities.found, x=Pathway.name)) +
geom_bar(stat="identity", width = 0.7)
p + coord_flip() + labs(x="reactome pathways") +
  scale_x_discrete(labels = function(x) str_wrap(x, width = 50))+
theme(text = element_text(size=15)) + ylim(0,60)

df10 <- read.csv("reactome cluster 10.csv")
df10 <- df10[1:10,c(2,3,5,7)]
df10 <- mutate(df10, "-log10(FDR)" = -log10(df10$Entities.FDR))
df10$Pathway.name <- factor(df10$Pathway.name, levels =
df10$Pathway.name[order(df10$Entities.FDR, decreasing = T)])

p2 <- ggplot(df10, aes(y=X.Entities.found, x=Pathway.name, colour=`-
log10(FDR)`, size = Entities.ratio)) + geom_point()
p2 + coord_flip() + labs(x="", y = "entities", colour="-log10(FDR)",
size="%entities") +
  scale_x_discrete(labels = function(x) str_wrap(x, width = 50)) + ylim(0,
40) + theme(text = element_text(size=15))
```
